# KaiC-dependent circadian positioning of RNA polymerase and ribosome in cyanobacteria

**DOI:** 10.1016/j.isci.2026.116630

**Published:** 2026-06-27

**Authors:** Lina Wang, Cuncun Qiao, Chi Zhao, Tao Zhu, Xuefeng Lu

**Affiliations:** 1 Key Laboratory of Photoelectric Conversion and Utilization of Solar Energy, Qingdao Institute of Bioenergy and Bioprocess Technology, Chinese Academy of Sciences, No. 189 Songling Road, Qingdao 266101, China; 2 Shandong Energy Institute, No. 189 Songling Road, Qingdao 266101, China; 3 Qingdao New Energy Shandong Laboratory, No. 189 Songling Road, Qingdao 266101, China; 4 College of Life Science, University of Chinese Academy of Sciences, Beijing 100049, China

**Keywords:** spatiotemporal compartmentalization, KaiC, RNA polymerase, ribosome, cyanobacteria

## Abstract

Circadian rhythms in *Synechococcus elongatus* PCC 7942 (*S. elongatus*) orchestrate global gene expression by regulating chromosomal DNA dynamics, transitioning between diffuse, and compacted states. While DNA dynamics have been extensively characterized, how the clock influences the spatial organization of transcriptional and translational machinery remains unclear. Using live-cell fluorescence imaging, we examined the circadian dynamics of RNA polymerase (RNAP) and ribosome localization in *S. elongatus*. We found that RNAPs colocalize with DNA during both diffuse and compacted states, whereas ribosomes colocalize with diffuse DNA but redistribute toward thylakoid membrane regions during DNA compaction. These rhythmic localization patterns persist under continuous light and dark conditions, remain stable within the tested time window following transcriptional or translational inhibition, and require a functional KaiC protein. Together, our findings support a model in which the cyanobacterial circadian clock coordinates RNAP, ribosome, and nucleoid spatial dynamics, revealing spatiotemporal organization as a layer of prokaryotic circadian regulation.

## Highlights

• RNAPs always colocalize with DNA regardless of nucleoid state
• Ribosomes re-localize to thylakoids during DNA compaction
• Rhythms persist under constant light/dark and during inhibitor treatment window
• *kaiC* deletion abolishes these spatial rhythms

## Graphical abstract

**Figure:**
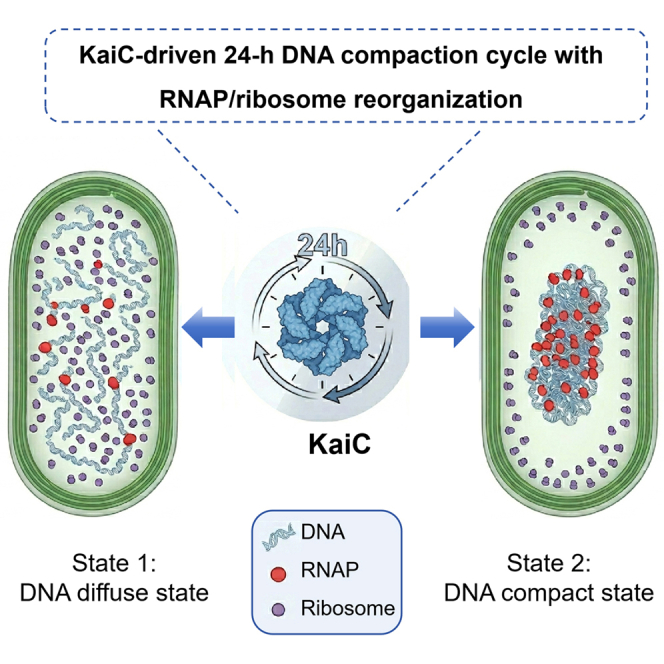

## Teaser

Biochemistry; Microbiology; Organizational aspects of cell biology

## Introduction

Circadian clocks have independently evolved in both prokaryotes and eukaryotes, enabling organisms to synchronize gene expression with daily environmental cycles such as light and temperature fluctuations.^1^^,^^2^^,^^3^ *Synechococcus elongatus* PCC 7942 (*S. elongatus*) serves as a foundational model for studying prokaryotic circadian systems, characterized by a minimalist yet robust oscillator.^4^^,^^5^^,^^6^^,^^7^ Approximately 64% of the *S. elongatus* transcriptome exhibits circadian oscillations, underscoring the broad regulatory reach of its endogenous clock.^8^ A key mechanism underlying this regulation is the rhythmic spatial organization of chromosomal DNA, which alternates between diffuse and compacted states to globally modulate gene expression.^9^^,^^10^^,^^11^ Pioneering work by Smith et al. demonstrated that the chromosome compaction rhythm is KaiC-dependent and proposed that it regulates global transcription by modulating promoter accessibility.^12^ However, despite this established chromosome compaction rhythm, it remains unclear how the core gene expression machinery, including RNA polymerases (RNAPs) and ribosomes, is spatially and temporally organized within this dynamic chromosomal landscape.

The spatial relationship between RNAPs, ribosomes, and DNA varies across bacterial species, reflecting fundamental constraints on gene expression. In non-circadian bacteria such as *Escherichia coli* (*E. coli*), freely diffusing RNAPs are distributed homogeneously throughout the nucleoid, while DNA-bound RNAPs localize preferentially to the nucleoid periphery.^13^^,^^14^ Ribosomes, in contrast, exhibit species-specific positioning that follows the nucleoid-to-cytoplasm (NC) ratio, a conceptual shift introduced by Gray et al., who showed that nucleoid size scales isometrically with cell size across bacterial phylogeny, forming a continuum of NC ratios rather than discrete organizational states.^15^ In species with low NC ratios (e.g., *E. coli* and *Bacillus subtilis*), ribosomes are largely excluded from the nucleoid interior, occupying nucleoid-free regions of the cytoplasm.^16^^,^^17^ This segregation is thought to minimize conflicts between transcription and translation, prevent replication-transcription collisions, and allow proper mRNA folding before ribosome engagement.^18^ In species with high NC ratios (e.g., *Caulobacter crescentus*), the nucleoid occupies a larger fraction of the cytoplasmic volume, forcing ribosomes to intermingle with DNA.^15^ What functional advantages might arise from translation occurring in proximity to DNA? In bacteria, transcription and translation are coupled in time and space: ribosomes can bind to the nascent mRNA while it is still being synthesized, a process known as co-transcriptional translation.^19^ This coupling enables rapid protein production, facilitates proper protein folding through domain-wise translation, and allows regulatory mechanisms such as attenuation and riboswitches to operate efficiently.^20^^,^^21^ When ribosomes are positioned near the nucleoid, either by passive confinement or active tethering, co-transcriptional translation is spatially favored, potentially enhancing gene expression efficiency.^22^

However, these studies primarily focus on static spatial patterns in non-circadian systems, leaving open questions about how such spatial organization changes over time in circadian organisms. In cyanobacteria such as *S. elongatus*, nucleoid size exhibits circadian oscillations between compact and diffuse states, suggesting that the NC ratio itself undergoes rhythmic fluctuations. However, despite this dynamic behavior, the spatial organization of RNAPs and ribosomes has only been characterized as static in non-circadian bacteria such as *Escherichia coli*, leaving it unclear whether these relationships are also subject to circadian regulation. These considerations raise two key questions: (1) what are the spatiotemporal dynamics of RNAPs and ribosomes in circadian bacteria such as *S. elongatus*? (2) Is their spatial organization directly regulated by the circadian clock, as in the case of nucleoid compaction, or is it a secondary outcome of transcriptional and translational activity?

To address these questions, we employed live-cell fluorescence imaging to monitor RNAP and ribosome dynamics in *S. elongatus* under light/dark (LD) cycles, constant light (LL), and constant darkness (DD) conditions. We demonstrate that RNAPs colocalized with chromosomal DNA in both diffuse and compacted states, whereas ribosomes colocalize with diffuse DNA but tend to accumulate at thylakoid membrane regions during DNA compaction, with a circadian (∼24-h) manner. These spatiotemporal patterns persist under LL or DD conditions and are unaffected by transcriptional or translational inhibition, suggesting them as clock-regulated processes rather than metabolic byproducts. Furthermore, deletion of the core clock gene *kaiC* abolishes these rhythms, suggesting that the circadian oscillator acts directly in the spatial regulation of the transcription and translation machinery. Our findings show that the spatial organization of ribosomes and RNAPs is dynamically regulated over the circadian cycle, alongside circadian changes in nucleoid organization that may affect the nucleocytoplasmic (NC) ratio, thereby extending Gray’s NC continuum model into the temporal dimension and providing new insights into circadian control of gene expression.

## Results

### Subcellular visualization of chromosomal DNA, RNAPs, and ribosomes in *S. elongatus*

To visualize the subcellular organization of chromosomal DNA, 4′,6-diamidino-2-phenylindole (DAPI) staining was used. To examine the spatial organization of RNAPs and ribosomes, we constructed three fluorescently tagged *S. elongatus* strains. First, a control strain (named strain NSIIeYFP) was generated by integrating the enhanced yellow fluorescent protein gene (*eyfp*) into the NSII neutral site under the control of the strong promoter P_*cpcB*_ (Figure S1A and Table S1). This strain enables visualization of freely diffusing eYFP within the intracellular environment. Subsequently, two additional strains were generated by replacing the chromosomal *rpoC2* (*Synpcc7942_1524*, encoding the RNAP β′ subunit) and *rpsB* (*Synpcc7942_2530*, encoding the S2 protein of the ribosomal 30S subunit) loci with *eyfp* fusion cassettes, resulting in RpoC2-eYFP and RpsB-eYFP strains, respectively (Figures S1B and S1C, and Table S1). These two strains allow visualization of the spatial dynamics of native RNAP and ribosome core or holoenzyme. Genotypic validation confirmed correct chromosomal integration in all three strains (Figures S1A–S1C), and western blot analysis verified expression of full-length fusion proteins without detectable degradation (Figure S1D). Furthermore, growth assays showed that all three strains grew comparably to *S. elongatus* wild-type (WT), indicating that the eYFP tags did not impair cell viability or physiology (Figure S1E).

To investigate the *in vivo* localization of chromosomal DNA, RNAPs, and ribosomes, we performed laser scanning confocal microscopy on live cells. DAPI-stained DNA was pseudo-colored orange via spectral unmixing to enhance contrast against a dark background, while imaging was conducted using the conventional blue emission channel (Figure S1F). Free eYFP, RpoC2-eYFP, and RpsB-eYFP were captured in the green channel, representing the spatial dynamics of free eYFP, RNAPs, and ribosomes, respectively (Figure S1F). Overlay of the orange pseudo-color (acquired in blue emission channel) and green (O/G) channels revealed spatial relationships between chromosomal DNA and RNAP or ribosome signals (Figure S1F). Additionally, autofluorescence from phycobiliproteins, detected in the red channel, delineated the thylakoid membrane architecture and served as a marker for cellular boundaries (Figure S1F).

### Circadian localization of RNAPs

**Figure 1:**
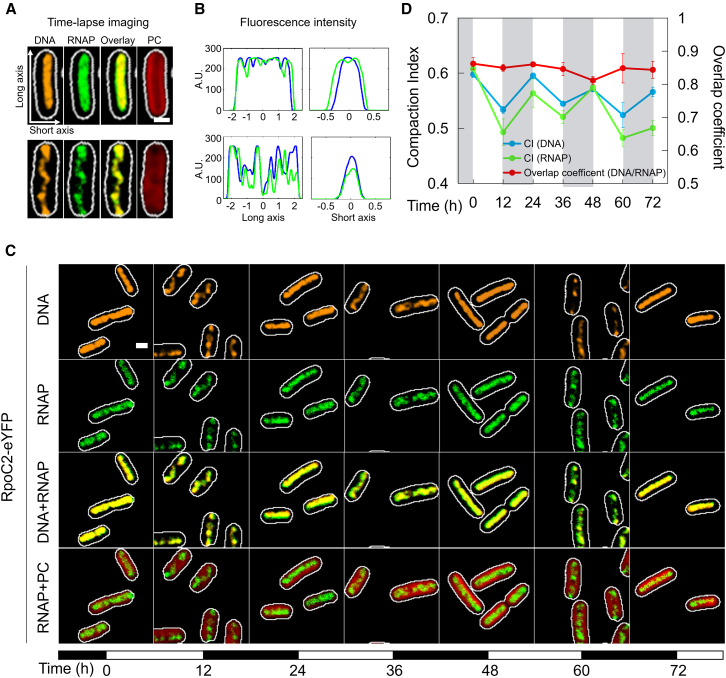
Subcellular organization of chromosomal DNA and RNAPs in *S. elongatus* (A) Representative spatial localization of RNAPs in *S. elongatus* during diffuse (upper, ZT 0) and compacted (lower, ZT 12) chromosomal DNA states. Fluorescence imaging shows chromosomal DNA (orange pseudo-color), RNAPs (green), overlay of DNA and RNAP signals (orange/green), and autofluorescence from phycobiliproteins associated with thylakoid membranes (red). (B) Axial (long axis) and radial (short axis) fluorescence intensity profiles through the cellular center, showing distributions of DNA (blue) and RNAP (green) signal. The profiles correspond to cells in (A). A.U., arbitrary units. (C) Circadian dynamics of RNAP localization across three consecutive 12 h:12 h light/dark (LD) cycles. Fluorescence images show chromosomal DNA (orange) and RNAPs (green) at the indicated times, with ZT 0 defined as the onset of light after 12 h dark entrainment period. White and black bars denote light and dark phases, respectively. Time points: 0/24/48/72 h correspond to the start of the day (dawn, ZT 0/24), 12/36/60 h correspond to the end of the day (dusk, ZT 12). Each time-course experiment was independently replicated three times with consistent results. Scale bars, 1 μm. (D) Quantitative analysis of circadian spatial dynamics during LD cycles, including DNA compaction index (blue), RNAP compaction index (green), and DNA/RNAP co-localization coefficients (red). Data points represent mean values from ≥100 cells per time point across three biological replicates. Gray backgrounds indicate dark phases (ZT 12–24). Error bars correspond to the standard deviation. Quantification was performed using ImageJ software.

Having visualized the DNA, RNAPs, and ribosomes in *S. elongatus*, we next explored the DNA and RNAP localization changes over the circadian cycle. Previous studies have reported that chromosomal DNA in *S. elongatus* undergoes circadian oscillations between diffuse and compacted states.^9^^,^^11^^,^^12^ Consistent with these findings, our confocal imaging revealed distinct diffuse and compacted states, as illustrated by two representative cells (Figure 1A, orange pseudo-color).

To ensure that the eYFP reporter itself does not bias localization patterns, we first examined the intracellular distribution of free eYFP in the control strain NSIIeYFP. Fluorescence imaging showed that free eYFP was distributed throughout the cytoplasm in both DNA diffusion and compaction states, with the exception of punctate regions that became visible during DNA compaction (Figure S2, red arrows). These punctate structures likely represent carboxysomes, which form biomolecular condensates via phase separation that exclude surrounding cytoplasmic proteins. Importantly, aside from these carboxysome-excluded regions, free eYFP showed no specific enrichment along the nucleoid or at the thylakoid membranes, confirming that the eYFP tag alone does not cf. subcellular targeting. We then examined RNAP localization using the RpoC2-eYFP strain (Table S1). We found that the RNAP organization also exhibited diffuse or compacted states, closely corresponding to the chromosomal DNA localization pattern (Figure 1A, green channel). Overlay of DNA and RNAP fluorescence signals further demonstrated a persistent spatiotemporal compartmentalization of RNAPs with chromosomal DNA in both diffuse and compacted states (Figure 1A, overlay channel).

To further support our observation, we extracted fluorescence intensity profiles along both the axial (long-axis) and radial (short-axis) directions from the two representative cells. These profiles confirmed a strong spatial correlation between RNAP and chromosomal DNA in both diffuse and compact states (Figure 1B). Quantification of Manders’ coefficients for the representative cells confirmed near-complete colocalization between RNAP and DNA in both diffuse (M1 = 0.999, M2 = 0.983) and compacted (M1 = 0.999, M2 = 0.998) states (Table S2). This spatial coupling indicates that RNA polymerase associated with the chromosome across distinct structural states of the nucleoid and suggests circadian modulation of the spatial organization of the transcription machinery rather than a binary switch in transcriptional activity.

To further investigate the spatiotemporal dynamics of RNAPs, we tracked the localization of both chromosomal DNA and RNAPs over three consecutive light/dark (LD) cycles. Cultures were entrained with a 12-h dark period before transfer to light. Samples were subsequently collected at 12-h intervals for 72 h at ZT 0 and ZT 12 (ZT is short for zeitgeber time). Fluorescence imaging revealed synchronized rhythmic patterns: diffuse distributions predominated at ZT 0 and 24, while compacted states peaked at ZT 12 (Figure 1C). Quantitative analysis using the compaction index (CI) and the overlap coefficients confirmed robust 24-h oscillations in DNA/RNAP condensation (CI peaks at ZT 0/24; troughs at ZT 12) with consistently high colocalization (overlap coefficient: 0.8–0.9; Figure 1D). Notably, the DNA-RNAP overlap coefficient exhibited remarkable stability throughout the circadian cycle, indicating that a substantial fraction of RNA polymerase remains associated with chromosomal DNA at all time points. This suggests that circadian regulation of gene expression is not achieved by large changes in the overall fraction of DNA-bound RNA polymerase, but more likely through changes in chromosomal architecture.

**Figure 2:**
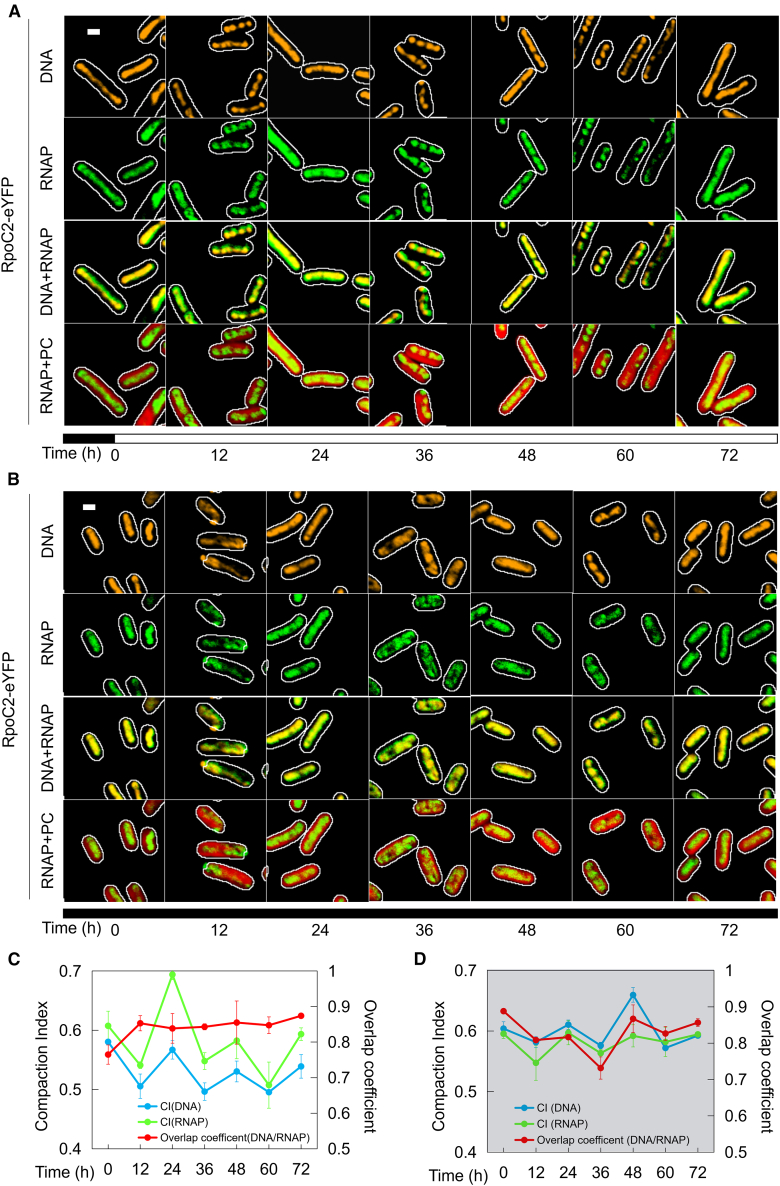
Subcellular distribution of chromosomal DNA and RNAPs in *S. elongatus* under constant conditions (A and B) Confocal images show representative fields of strain RpoC2-eYFP at indicated time points during LL (A) and DD (B) cycles. Cultures were entrained by 12 h darkness prior to release into LL/DD at LL 0/DD 0. Time points 0, 24, 48, and 72 h correspond to subjective dawn, while timepoints 12, 36, and 60 h correspond to subjective dusk. Orange: DAPI-stained DNA; green: RNAPs; red, autofluorescence from phycobiliproteins. Images represent >90% of the cells observed for each time point. Scale bars, 1 μm. (C and D) Quantitative analysis of chromosomal DNA compaction index (CI) and DNA-RNAP colocalization (overlap coefficient) over time during LL (C) and DD (D) cycles. Data represent mean ± SD from at least 300 cells, analyzed using ImageJ software. Gray shaded areas indicate time periods without light exposure.

To determine whether the observed rhythmic spatial coordination between RNAP and DNA persists endogenously, we examined cells following entrainment under constant conditions. After the entrainment with a 12-h darkness, cultures were transferred to continuous light (LL) or continuous darkness (DD), and samples were collected every 12 h. Fluorescence imaging showed that RNAP localization remained tightly coupled to chromosomal DNA organization, with diffuse distributions at time points 0/24/48/72 h, and compaction at time points 12/36/60 h (Figures 2A and 2B). Quantitative analysis revealed sustained ∼24-h oscillations in the CI values of both DNA and RNAP (Figures 2C and 2D), alongside consistently high spatial colocalization (overlap coefficient: 0.7–0.9). To capture finer temporal dynamics, we tracked individual cells at 4-h intervals during the first circadian cycle after entrainment. These fluorescence imaging revealed a gradual and continuous progression between diffuse and compacted states under all three conditions (LD, LL, and DD), indicating that the spatial oscillations are not abrupt but develop progressively over time (Figure S3). Collectively, these findings reveal that RNAPs in *S. elongatus* undergo a robust 24-h rhythm of spatial compartmentalization, dynamically alternating between diffuse and compacted DNA states in tight coordination with chromosomal architecture, and maintained independently of external light input.

### Circadian localization of ribosomes

**Figure 3:**
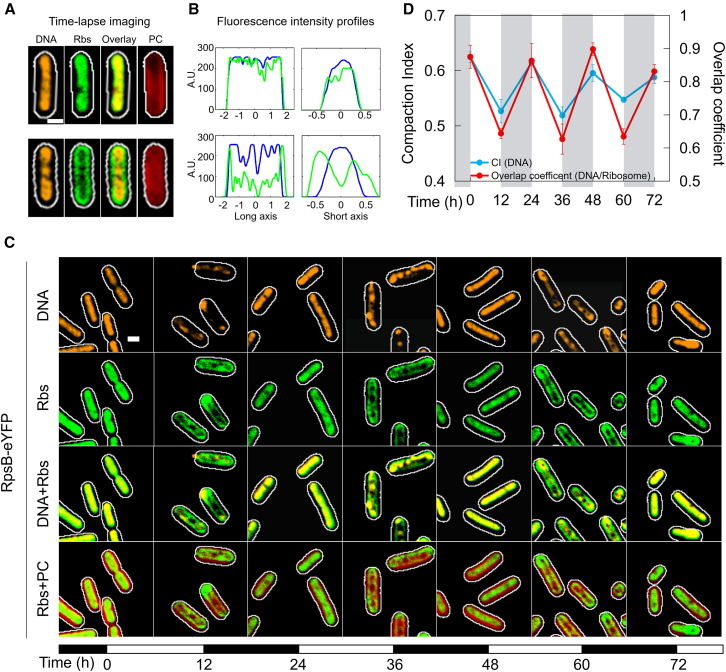
Subcellular organization of chromosomal DNA and ribosomes in *S. elongatus* (A) Representative spatial localization of ribosomes in *S. elongatus* during diffuse (upper, ZT 0) and compacted (lower, ZT 12) chromosomal DNA states. Fluorescence imaging shows chromosomal DNA (orange pseudo-color), ribosomes (green), overlay of DNA, and ribosome signals (orange/green), and autofluorescence from phycobiliproteins associated with thylakoid membranes (red). (B) Axial (long axis) and radial (short axis) fluorescence intensity profiles through the cellular center, showing distributions of DNA (blue) and ribosome (green) signal. The profiles correspond to cells in (A). A.U., arbitrary units. (C) Circadian dynamics of ribosome localization across three consecutive 12 h:12 h light/dark (LD) cycles. Fluorescence images show chromosomal DNA (orange) and ribosomes (green) at the indicated times, with ZT 0 defined as the onset of light after 12 h dark entrainment period. White and black bars denote light and dark phases, respectively. Time points: 0/24/48/72 h correspond to the start of the day (dawn, ZT 0/24), 12/36/60 h correspond to the end of the day (dusk, ZT 12). Each time course experiment was independently replicated three times with consistent results. Scale bars, 1 μm. (D) Quantitative analysis of circadian spatial dynamics during LD cycles, including DNA compaction index (blue) and DNA/ribosome co-localization coefficients (red) during LD cycles. Data points represent mean values from ≥100 cells per time point across three biological replicates. Gray backgrounds indicate dark phases (ZT 12–24). Error bars correspond to the standard deviation. Quantification was performed using ImageJ software.

Having established the spatiotemporal dynamics of RNAPs, we next investigated whether ribosomes exhibit similar circadian patterning using the RpsB-eYFP reporter strain (Table S1). Confocal imaging under LD conditions revealed a distinct circadian compartmentalization of ribosomes relative to chromosomal architecture. At dawn (ZT 0), when chromosomal DNA adopted a diffuse conformation, ribosomes were homogenously distributed throughout the DNA-rich regions (Figure 3A upper). Strikingly, upon DNA compaction at dusk (ZT 12), ribosomes underwent marked spatial redistribution, relocating predominantly to subcellular regions proximal to the thylakoid membranes, indicating a reorganization tied to DNA compaction (Figure 3A lower). Manders’ coefficients for the representative cells also revealed a marked decrease in ribosome-DNA colocalization upon DNA compaction (M1: from 0.999 to 0.857; M2: from 0.992 to 0.843) (Table S2).

Axial and radial fluorescence intensity profiling corroborated this spatial redistribution. During DNA-diffuse phases (ZT 0/24), ribosomal signals were closely aligned with chromosomal DNA across the long and short axes of the cell (Figure 3B upper). Conversely, during DNA compaction (ZT 12), intensity profiles demonstrated pronounced enrichment of ribosomal fluorescence at the cell periphery (Figure 3B lower). Population-level short-axis profiling (>50 cells) further confirmed this circadian repositioning: ribosomal signals shifted from nucleoid-associated localization at ZT 0 to enrichment at the cell periphery at ZT 12 (Figures 3B and S4). Crucially, this peripheral enrichment overlapped with signals from the thylakoid membrane (Figure S4), indicating spatial proximity between ribosomes and the thylakoid membranes during chromosomal compaction, though direct physical interaction cannot be inferred from colocalization alone.

Longitudinal tracking across multiple LD cycles confirmed that ribosome localization is strongly synchronized with the circadian cycle. Diffuse distributions persisted at dawn (ZT 0/24), whereas membrane-proximal enrichment consistently recurred at dusk (ZT 12) (Figure 3C). Corresponding overlap coefficient analysis revealed this rhythmic fluctuation, with higher values (0.8–0.9) during diffuse phases and lower values (0.6–0.7) during chromosomal compaction, reflecting periodic spatial segregation of ribosomes, and DNA (Figure 3D).

**Figure 4:**
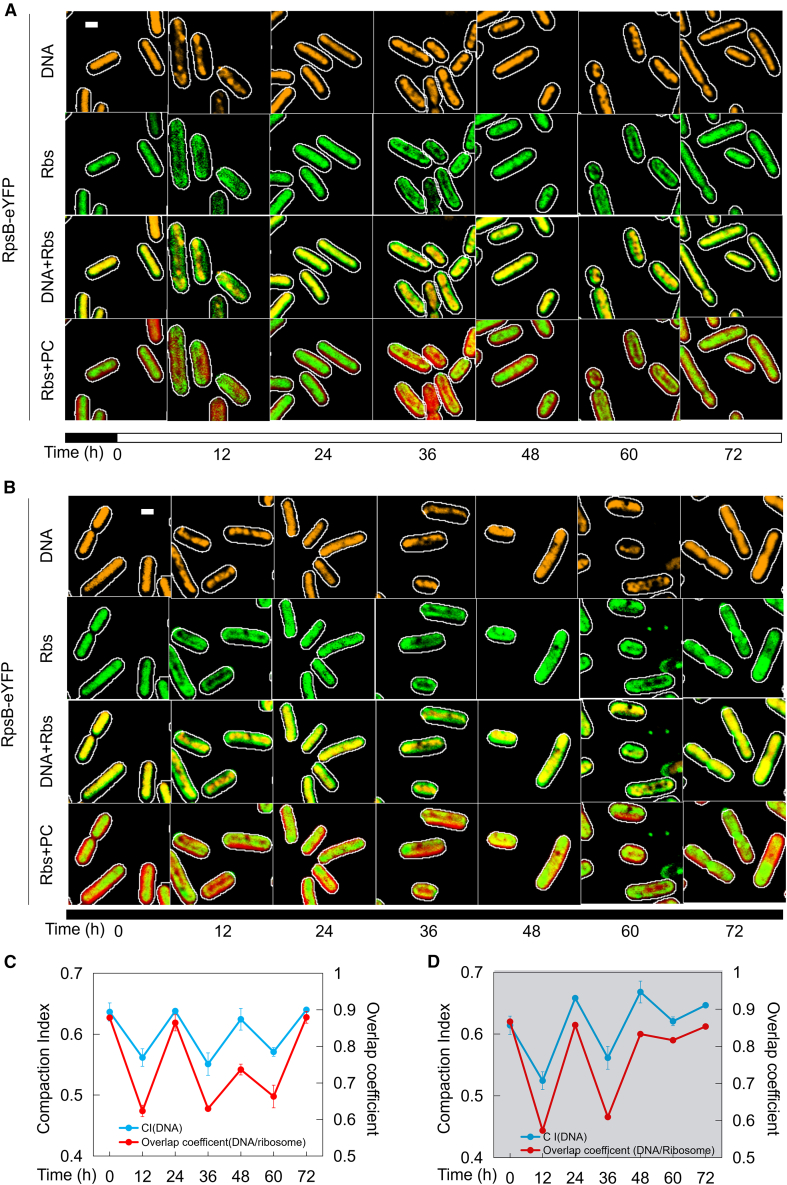
Subcellular distribution of chromosomal DNA and ribosomes in *S. elongatus* under constant conditions (A and B) Confocal images showing representative fields of strain RpsB-eYFP at indicated time points during LL (A) and DD (B) cycles. Cultures were entrained by 12 h darkness prior to release into LL/DD at LL 0/DD 0. Time points 0, 24, 48, and 72 h correspond to subjective dawn, while time points 12, 36, and 60 h correspond to subjective dusk. Orange: DAPI-stained DNA; green: ribosomes; red, autofluorescence from phycobiliproteins. Images represent >90% of the cells observed at each time point. Scale bars, 1 μm. (C and D) Quantitative analysis of chromosomal DNA compaction index (CI) and DNA-ribosome colocalization (overlap coefficient) over time during LL (C) and DD (D) cycles. Data represent mean ± SD from at least 300 cells, analyzed using ImageJ software. Gray shaded areas indicate time periods without light exposure.

To assess whether this rhythmic pattern is intrinsic, we examined entrained cells under constant light (LL) and darkness (DD), respectively. Ribosomal redistribution rhythms persisted autonomously, with diffuse localization at subjective dawn (0/24/48/72 h) and peripheral enrichment at subjective dusk (12/36/60 h) (Figures 4A and 4B). Corresponding overlap coefficients continued to oscillate (LL: 0.75–0.85 in diffuse to 0.55–0.65 in compacted states; DD: 0.70–0.80 in diffuse to 0.50–0.60 in compacted states), indicating that this spatial rhythm is maintained independently of external light cues (Figures 4C and 4D). Furthermore, 4-h interval imaging of individual cells showed gradual, coordinated transitions between diffuse and compacted states under LD, LL, and DD, indicating that the spatial transitions proceed progressively rather than abruptly, consistent with coordinated circadian regulation (Figure S5).

Together, these results demonstrate that ribosomes undergo circadian-driven spatiotemporal compartmentalization in *S*. *elongatus*, alternating between chromosomal DNA-associated and membrane-proximal states. This dynamic partitioning suggests a clock-controlled mechanism that integrates ribosome localization with chromosomal architecture.

### RNAP/ribosome compartmentalization is independent of transcription

As described previously, the spatiotemporal distributions of chromosomal DNA, RNAPs, and ribosomes in *S. elongatus* exhibit a robust 24-h periodic rhythm. Despite clear evidence that chromosomal DNA spatial dynamics are governed by the circadian clock, the mechanisms controlling RNAP and ribosome compartmentalization rhythms remain poorly understood. One proposed mechanism is transertion, the simultaneous processes of transcription, translation, and membrane insertion of nascent proteins, which could potentially organize macromolecular complexes.^23^^,^^24^

**Figure 5:**
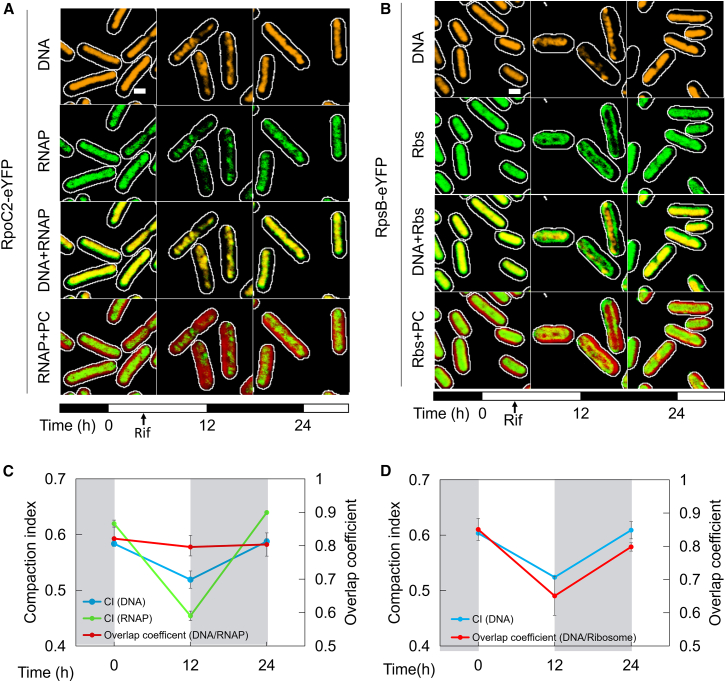
Effects of rifampicin (Rif) treatment on the subcellular organization of the chromosomal DNA, RNAPs, and ribosomes in *S. elongatus* (A) RNAP localization under Rif treatment (200 μg/mL). Fluorescence images show DNA (orange pseudo-color) and RNAPs (green) at ZT 0, 12, and 24. ZT 0 is defined as light-onset after 12 h dark entrainment (dawn). Rif was applied at ZT 4. White/black bars denote light/dark phases. Experiments were independently replicated three times. Scale bars: 1 μm. (B) Ribosome localization under Rif treatment (200 μg/mL). Images show DNA (orange pseudo-color) and ribosomes (green) at ZT 0, 12, and 24. ZT 0 is defined as light-onset after 12 h dark entrainment (dawn). Rif was applied at ZT 4. White/black bars denote light/dark phases. Experiments were independently replicated three times. Scale bars: 1 μm. (C) Quantitative analysis of DNA compaction (blue), RNAP compaction (green), and DNA/RNAP co-localization (red) under the treatment of Rif. Gray backgrounds indicate dark phases (ZT 12–24). Data points represent means from ≥100 cells/time point across three biological replicates. Error bars correspond to the standard deviation. Analysis performed with ImageJ. (D) Quantitative analysis of DNA compaction (blue) and DNA/ribosome co-localization (red) under the treatment of Rif. Gray backgrounds indicate dark phases (ZT 12–24). Data points represent means from ≥100 cells/time point across three biological replicates. Error bars correspond to the standard deviation. Analysis performed with ImageJ.

To investigate this, we inhibited transcription using rifampicin (Rif), an antibiotic that blocks RNAP-mediated transcription initiation by obstructing the RNA exit channel.^25^^,^^26^ In *S. elongatus*, treatment with Rif (200 μg/mL, 4 h) has been reported to inhibit over 95% of RNA synthesis while maintaining cell viability for at least 48 h.^27^^,^^28^^,^^29^ Based on this, RpoC2-eYFP and RpsB-eYFP strains were treated with Rif (200 μg/mL) at ZT 4 under LD conditions following entrainment (12 h dark → light at ZT 0). Growth was monitored over 120 h, and viability was assessed 36 h after entrainment. Consistent with previous studies, Rif treatment resulted in a measurable decline in growth after 48 h, while cell viability remained largely unaffected between wild-type and Rif-treated cells during the 36 h (Figure S6). Imaging at ZT 0, 12, and 24 revealed that RNAPs maintained colocalization with chromosomal DNA in both diffuse (ZT 0/24) and compacted (ZT 12) states, and ribosomes continued to associate with DNA at ZT 0/24 but redistributed to thylakoid membrane regions at ZT 12 (Figures 5A and 5B). CI analysis confirmed the persistence of 24-h oscillations in both DNA and RNAP organization, with peaks at ZT 0/24 and troughs at ZT 12 (Figure 5C). Moreover, the overlap coefficient between DNA and ribosomes continued to exhibit characteristic peak-to-valley oscillations with a ∼24-h period (Figure 5D). Importantly, phycobiliprotein autofluorescence, which delineates thylakoid membrane architecture, remained intact under Rif treatment throughout the imaging time course (Figures 5A and 5B), indicating that antibiotic treatment did not disrupt membrane integrity within the experimental time frame. These findings demonstrate that, within the 36-h experimental window, transcriptional inhibition by Rif does not perturb the spatiotemporal compartmentalization of RNAP and ribosome. Thus, transcription-coupled processes, such as transertion, are unlikely to be the primary drivers of this compartmentalization rhythm over the time frame examined.

### RNAP/ribosome compartmentalization is independent of translation

**Figure 6:**
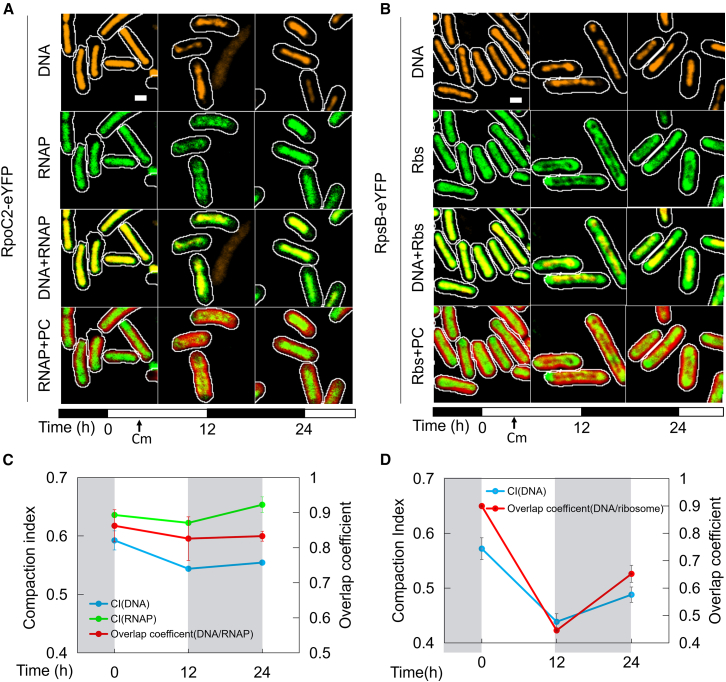
Effects of chloramphenicol (Cm) treatment on the subcellular organization of the chromosomal DNA, RNAPs, and ribosomes in *S. elongatus* (A) RNAP localization under Cm treatment (200 μg/mL). Fluorescence images show DNA (orange pseudo-color) and RNAPs (green) at ZT 0, 12, and 24. ZT 0 is defined as light-onset after 12 h dark entrainment (dawn). Cm was applied at ZT 4. White/black bars denote light/dark phases. Experiments were independently replicated three times. Scale bars: 1 μm. (B) Ribosome localization under Cm treatment (200 μg/mL). Images show DNA (orange pseudo-color) and ribosomes (green) at ZT 0, 12, and 24. ZT 0 defined as light-onset after 12 h dark entrainment (dawn). Cm was applied at ZT 4. White/black bars denote light/dark phases. Experiments independently replicated three times. Scale bars: 1 μm. (C) Quantitative analysis of DNA compaction (blue), RNAP compaction (green), and DNA/RNAP co-localization (red) under the treatment of Cm. Gray backgrounds indicate dark phases (ZT 12–24). Data points represent means from ≥100 cells/time point across three biological replicates. Error bars correspond to the standard deviation. Analysis performed with ImageJ. (D) Quantitative analysis of DNA compaction (blue) and DNA/ribosome co-localization (red) under the treatment of Cm. Gray backgrounds indicate dark phases (ZT 12–24). Data points represent means from ≥100 cells/time point across three biological replicates. Error bars correspond to the standard deviation. Analysis performed with ImageJ.

To further evaluate the role of transertion in the circadian compartmentalization of RNAPs and ribosomes, we inhibited translation using chloramphenicol (Cm), an antibiotic that targets the 50S ribosomal subunit and blocks peptide bond formation.^30^ In *S. elongatus*, Cm concentrations of 100–800 μg/mL have been validated for effective protein synthesis inhibition.^31^^,^^32^ Based on this, we treated RpoC2-eYFP and RpsB-eYFP strains with 200 μg/mL Cm at ZT 4 (post-entrainment) and subsequently monitored cell growth and cell viability. Consistent with previous studies, cell viability remained stable up to 36 h under Cm treatment (Figure S6). Imaging at ZT 0, 12, and 24 showed that RNAPs and ribosomes retained their spatial patterns, indistinguishable from untreated cells (Figures 6A and 6B). CI analysis confirmed that DNA and RNAP oscillations persisted with peaks at ZT 0/24 and troughs at ZT 12 (Figure 6C), and DNA-ribosome overlap coefficients continued to oscillate in resonance with the LD cycles (Figure 6D). Short-axis profiling further supported these results, revealing rhythmic ribosome-thylakoid membrane coupling under Cm treatment (Figure S7). At ZT 0, ribosome peaks were spatially separated from thylakoid membrane signals; at ZT 12, ribosome signals colocalized with the thylakoid membrane, indicating a rhythmic coordination of ribosome positioning with membrane compartments. This spatiotemporal pattern mirrored untreated controls, suggesting that translational inhibition does not disrupt the circadian spatiotemporal compartmentalization of RNAPs and ribosomes within the 36-h experimental window. Collectively, these results indicate that the transertion hypothesis is unlikely to be the primary mechanism driving RNAP and ribosome localization under the conditions tested.

### KaiC-dependent circadian compartmentalization of the transcriptional and translational machineries

**Figure 7:**
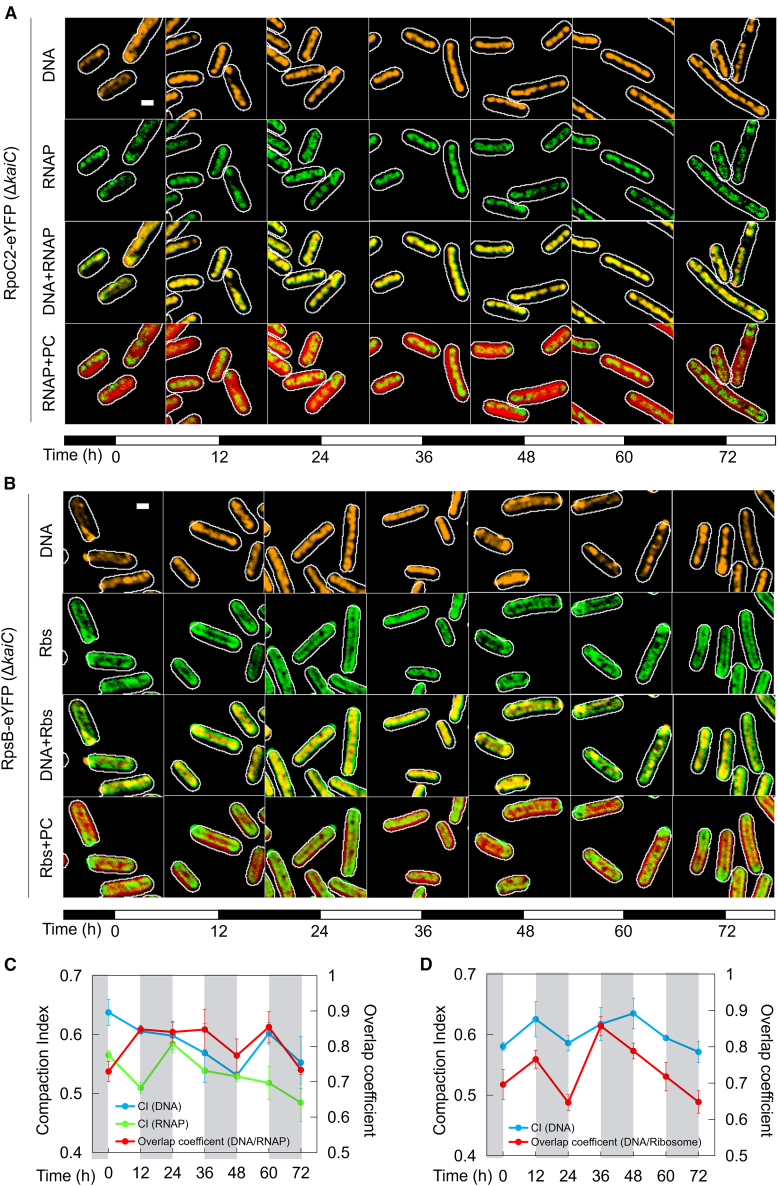
Subcellular organization of chromosomal DNA, RNAPs, and ribosomes in the *kaiC*-null strains under LD cycles (A) RNAP localization in *kaiC*-null strains. Fluorescence images show chromosomal DNA (orange pseudo-color) and RNAPs (green) at the indicated times, with ZT 0 defined as the onset of light after 12 h dark entrainment period. White and black bars denote light and dark phases, respectively. Time points: 0/24/48/72 h (the start of the day, dawn, ZT 0/24) and 12/36/60 h (the end of the day, dusk, ZT 12). Each time course experiment was independently replicated three times with consistent results. Scale bars, 1 μm. (B) Ribosomes localization in *kaiC*-null strains. Fluorescence images show chromosomal DNA (orange pseudo-color) and ribosomes (green) at the indicated times, with ZT 0 defined as the onset of light after 12 h dark entrainment period. White and black bars denote light and dark phases respectively. Time points: 0/24/48/72 h (the start of the day, dawn, ZT 0/24) and 12/36/60 h (the end of the day, dusk, ZT 12). Each time course experiment was independently replicated three times with consistent results. Scale bars, 1 μm. (C) Quantitative analysis of DNA compaction (blue), RNAP compaction (green), and DNA/RNAP co-localization (red) in the *kaiC*-null strains during LD cycles. Gray backgrounds indicate dark phases (ZT 12–24). Data points represent means from ≥100 cells/time point across three biological replicates. Error bars correspond to the standard deviation. Analysis performed with ImageJ. (D) Quantitative analysis of DNA compaction (blue) and DNA/ribosome co-localization (red) in the *kaiC*-null strains during LD cycles. Gray backgrounds indicate dark phases (ZT 12–24). Data points represent means from ≥100 cells/time point across three biological replicates. Error bars correspond to the standard deviation. Analysis performed with ImageJ.

Previous studies have shown that the spatiotemporal organization of chromosomal DNA in *S. elongatus* is regulated by KaiC-containing protein complexes, the core components of the cyanobacterial circadian clock.^9^^,^^11^^,^^12^ Given that transcription and translation follow a hierarchical flow from DNA to RNA to protein,^33^ and that chromosomal DNA organization is under circadian control, we hypothesized that the circadian clock coordinates RNAP and ribosome dynamics, directly or indirectly, by modulating the spatial organization of chromosomal DNA. To test this, we constructed two *S. elongatus* mutant strains, RpoC2-eYFP (Δ*kaiC*) and RpsB-eYFP (Δ*kaiC*), in which the *kaiC* gene was successfully deleted by genotypic analysis (Figure S8). After the entrainment of 12-h darkness, we monitored the spatiotemporal distributions of chromosomal DNA, RNAPs, and ribosomes in the absence of KaiC. Consistent with previous findings,^12^ deletion of *kaiC* abolished the periodic transitions between diffuse and compacted chromosomal DNA states, resulting in arrhythmic and heterogeneous DNA dynamics on a circadian timescale (Figures 7A and 7B). CI analysis across ≥100 cells per time point further confirmed the loss of 24-h peak-to-valley oscillations in DNA organization while revealing irregular fluctuations in compaction over time (Figures 7C and 7D). Importantly, both diffuse and compact nucleoid morphologies were observed at different time points in the Δ*kaiC* population, indicating that the chromosome is not locked in a constitutively compact state but instead fluctuates irregularly.

In line with the disrupted DNA dynamics, both RNAPs and ribosomes also lost their circadian rhythmicity in the *kaiC*-null strains (Figure 7). Despite this arrhythmicity, the spatial localization of RNAPs and ribosomes remained closely linked to the DNA state. Specifically, RNAP-DNA overlap coefficients remained consistently high (∼0.8) in the *kaiC*-null strain, indicating sustained colocalization regardless of circadian disruption (Figure 7C). Similarly, ribosomes continued to colocalize with diffuse chromosomal DNA (overlap coefficient: 0.8–0.9) and relocalized to membrane-proximal regions during DNA compaction (Figure 7D). To quantitatively evaluate rhythmicity, we also fitted all CI and overlay datasets to a 24-h cosine model. Wild-type cells showed strong periodic behavior, whereas the Δ*kaiC* related strains exhibited markedly reduced goodness-of-fit and non-significant *p* values across all parameters (Table S3), indicating loss of coherent circadian rhythmicity. Together, these findings demonstrate that the circadian rhythm governed by KaiC orchestrates the spatiotemporal compartmentalization of DNA, RNAPs, and ribosomes, thereby regulating transcription and translation to drive gene expression in cyanobacteria.

## Discussion

Our study reveals that the cyanobacterium *S*. *elongatus* exhibits a robust 24-h rhythm in the subcellular organization of its core gene expression machinery: chromosomal DNA, RNAPs, and ribosomes. Crucially, our findings identify the circadian clock as a key factor associated with these spatiotemporal patterning, as the rhythm persists under both continuous light and dark conditions and is abolished in *kaiC*-null mutants. Remarkably, this rhythmic spatial organization was maintained during the tested time window following transcriptional or translational inhibition, attributable to the post-translational KaiC phosphorylation cycle and the exceptional stability of KaiC.^27^^,^^31^

Previous studies in non-circadian bacteria have shown that RNAPs typically localize near the nucleoid, while ribosomes exhibit species-dependent patterns of colocalization or segregation from DNA.^34^^,^^35^^,^^36^ However, such observations were largely based on fixed-cell imaging, limiting the ability to resolve dynamic behaviors. Our work introduces a critical temporal dimension to these observations, uncovering a rhythmic, clock-regulated reorganization of RNAPs and ribosomes in *S*. *elongatus*. This dynamic redistribution, governed by the Kai-based oscillator, represents a previously unknown layer of temporal control over bacterial gene expression machinery. Our data align with the concept proposed by Gray et al.,^15^ linking nucleoid size and ribosome organization to the nucleoid-to-cytoplasm (N/C) ratio. We extend this model by demonstrating that rhythmic, clock-driven changes in nucleoid compaction within a single species directly dictate the spatiotemporal segregation of ribosomes: when the nucleoid is diffuse (high effective N/C ratio), ribosomes colocalize with DNA, whereas upon nucleoid compaction (low effective N/C ratio), ribosomes are excluded and enriched at the thylakoid membranes.

Building on these findings, we propose a model in which the circadian clock acts as a master regulator of spatiotemporal compartmentalization, dynamically partitioning the transcriptional and translational machineries in cyanobacteria. This clock-driven spatial organization likely serves to optimize cellular function by rhythmically enhancing transcription-translation coupling in a phase-specific manner. For instance, during subjective day, the enrichment of ribosomes at thylakoid membranes could facilitate the co-translational assembly of photosynthetic complexes. Transcriptomic analyses in cyanobacteria have shown that gene expression is broadly organized into dawn- and dusk-peaking cohorts rather than a uniform daytime maximum, with many photosynthesis-related genes exhibiting peak expression around subjective dawn, such as *psbAII* (encoding a photosystem II core protein), the *atp* operon (encoding ATP synthase subunits).^28^ The spatial redistribution of ribosomes observed here may therefore align translation capacity with these phase-specific transcriptional programs. Conversely, during subjective night, the colocalization of RNAPs and ribosomes within the nucleoid may prioritize the synthesis of housekeeping proteins, thereby conserving energy by minimizing mRNA trafficking and reducing non-essential processes like photosynthesis—a strategy consistent with the reported decline in glycogen reserves at night.^37^ Thus, circadian compartmentalization appears to tightly couple temporal gene regulation with spatial organization and metabolic state. From an evolutionary standpoint, this mechanism likely provides an adaptive advantage by enabling proactive optimization of resource allocation in anticipation of daily environmental cycles. More broadly, our findings suggest a form of spatial organization in prokaryotes that is achieved through dynamic, clock-associated redistribution of cellular components rather than membrane-bound compartmentalization.

Despite these insights, the molecular mechanisms linking the KaiC phosphorylation cycle to large-scale chromosomal reorganization and macromolecular compartmentalization remain elusive. Here, we propose a working model in which KaiC regulates nucleoid architecture by modulating chromosome supercoiling and nucleoid-associated protein activity in a circadian manner, thereby coupling the core oscillator to chromosomal organization. Specifically, the “oscilloid model” posits that KaiC-containing complexes could associate with the chromosome and rhythmically regulate negative supercoiling density.^9^^,^^10^ Increased negative supercoiling promotes DNA condensation, whereas relaxation favors a more diffuse chromosomal state, providing a direct physical link between the circadian clock and nucleoid architecture. At the next level, changes in DNA supercoiling are known to globally reshape transcriptional landscapes by modulating promoter accessibility and RNA polymerase initiation efficiency, thereby potentially redistributing transcriptional activity across the chromosome.^8^^,^^11^ Such transcriptional changes may, in turn, influence downstream translational outputs through altered mRNA availability and ribosome engagement. Together, these effects on DNA topology, transcription, and translation suggest possible pathways through which KaiC-driven oscillations could be associated with changes in chromosomal organization and gene expression dynamics, without implying direct mechanistic causality between each layer.

Future work should employ techniques such as Hi-C (high-throughput chromosome conformation capture) to elucidate how the clock orchestrates circadian-phase-specific chromosomal architecture and its relationship to RNAP and ribosome positioning. Integrating spatial transcriptomics with Hi-C could further reveal the three-dimensional gene expression landscape and regulatory loops between circadian chromatin domains and the gene expression machinery. We also note that all localization analyses were performed using eYFP fusions, and while the free eYFP control and distinct patterns across different fusions argue against tag-induced artifacts, confirmation with an orthogonal fluorescent protein tag (e.g., ZsGreen, GFP, or eCFP) would further strengthen these conclusions in future studies. Finally, because light-dark transitions provide a primary environmental input that directly drives cellular metabolism, it is important to assess their potential contribution to spatial organization. Thus, investigating whether metabolic cycles can independently organize the transcriptional and translational machinery, particularly when uncoupled from the circadian clock, will help clarify the relative contributions of metabolic and clock-dependent regulation.

### Limitations of the study

Several limitations should be considered when interpreting our results. First, all localization analyses were performed using fluorescent protein fusions, which may not fully recapitulate the native behavior of the tagged proteins. Second, although we show that rhythmic spatial patterns persist under continuous light and dark conditions and within the tested time window following transcriptional or translational inhibition, these perturbations do not fully eliminate pre-existing protein pools, and therefore residual protein stability may contribute to the observed signals. Third, while our data support a model linking KaiC-dependent circadian dynamics to spatial organization of gene expression machinery, the direct molecular mechanisms underlying this coupling remain to be experimentally established. Finally, this study was performed in a single model cyanobacterium, and whether similar spatiotemporal organization exists in other prokaryotic systems remains to be determined.

## Resource availability

### Lead contact

Requests for further information and resources should be directed to and will be fulfilled by the lead contact, Xuefeng Lu (lvxf@qibebt.ac.cn).

### Materials availability

All bacterial and cyanobacterial derivatives generated in this study are available from the lead contact with a completed materials transfer agreement.

### Data and code availability

• All data reported in this article and its analysis is available from the lead contact upon request.
• This study did not generate new code.
• Any additional information required to reanalyze the data reported in this paper is available from the lead contact upon request.

## Acknowledgments

This work was supported by the 10.13039/100014103Key Research and Development Program of Shandong Province, China (2025CXPT029), the Strategic Priority Research Program of Chinese Academy of Sciences (XDB1290103), the 10.13039/501100012166National Key Research and Development Program of China, China (2021YFA0909700 and 2024YFB4106501), the 10.13039/501100001809National Natural Science Foundation of China, China (32570172), the QIBEBT International Cooperation Project (QIBEBT ICP202301), the Qingdao Science and Technology Benefit People Demonstration Guide Special Project (24-1-8-xdny-17-nsh), the Shandong Marine Science and Technology Achievements Transfer and Commercialization Center “Open Bidding and Leadership Program” (2023JBGS02), and the Shandong Taishan Scholarship (X.L.). The authors would like to acknowledge Chunpeng Yang for her assistance in conducting the confocal microscopy and Prof. Xudong Xu for providing the plasmid pRL446.

## Author contributions

X.L. and T.Z. conceived the study. T.Z. and L.W. designed and performed the experiments. L.W., T.Z., and C.Q. analyzed the data. L.W. and T.Z. wrote the manuscript. L.W., T.Z., C.Q., C.Z., and X.L. reviewed and edited the manuscript. X.L. and T.Z. acquired funding and supervised the project.

## Declaration of interests

The authors declare no competing interests.

## STAR★Methods

### Key resources table

**Table:** 

REAGENT or RESOURCE	SOURCE	IDENTIFIER
**Antibodies**		

Mouse monoclonal anti-GFP-tag antibody	TransGen	Cat# HT801; RRID:AB_2922385
Goat anti-mouse lgG/AP antibody	Thermo Fisher	Cat# PA1-28755; RRID:AB_10978120

**Bacterial and virus strains**		

*Synechococcus elongates* PCC 7942	Lab substrain, originated from the Pasteur Culture Collection of Cyanobacteria	PCC 7942
NSII:eYFP	This paper	N/A
RpoC2-eYFP	This paper	N/A
RpsB-eYFP	This paper	N/A
RpoC2-eYFP (Δ*kaiC*)	This paper	N/A
RpsB-eYFP (Δ*kaiC*)	This paper	N/A

**Chemicals, peptides, and recombinant proteins**		

4′,6-diamidino-2-phenylindole	Solarbio	Cat# ID2250; CAS: 28718-90-3
Rifampicin	Solarbio	Cat# R8011; CAS:13292-46-1
Chloramphenicol	Solarbio	Cat# C8050; CAS: 56-75-7

**Critical commercial assays**		

ClonExpress MultiS Kit	Vazyme	Cat# C113-01

**Oligonucleotides**		

Primers, see Table S1	This paper	N/A

**Recombinant DNA**		

Plasmids, see Table S1	This paper	N/A

**Software and algorithms**		

ImageJ	Fiji	https://imagej.nih.gov/ij/
GraphPad Prism v11.0.0	GraphPad Software, LLC	https://www.graphpad.com/
FV10-ASW 4.0 software	Olympus	www.olympus-lifescience.com

### Experimental model and study participant details

#### Cyanobacterial strains and culture conditions

*S. elongatus*, originally obtained from the Pasteur Culture Collection of Cyanobacteria, and its derivative strains were cultured in standard BG11 liquid medium supplemented with appropriate antibiotics: 10 μg/mL gentamycin, 15 μg/mL kanamycin, and 10 μg/mL spectinomycin. The full list of strains used in this study is provided in Table S1. Except where indicated, all strains were inoculated at an initial optical density OD_730_ of 0.05 in 50 mL Erlenmeyer flasks containing 30 mL of fresh BG11 medium. Cultures were then incubated at 30 °C under constant illumination (30–40 μmol photons m^−2^ s^−1^) until reaching an OD_730_ of 0.15–0.2. For growth on solid medium, BG11 was supplemented with 1.4% agar. Cell density was measured at OD_730_ using a UV-visible spectrophotometer (PGENERAL, China).

### Method details

#### Cloning and strain construction

All plasmids, strains, and primers used in this study are listed in Table S1, with detailed descriptions provided in this section. For the construction of control strain NSIIeYFP, the P_*cpcB*_ promoter, e*yfp* cassette and gentamicin resistance gene (Gm^r^) were amplified from *S*. *elongatus* genomic DNA, pLAU53 (Table S1) and pHM005,^38^ respectively. These fragments were assembled via fusion PCR and cloned into the *Eco*91 I digested, T4 DNA polymerase-blunted pQL224n integration vector,^39^ resulting in plasmid pQL233. Transformation of pQL233 into *S. elongatus* generated the control strain NSIIeYFP.^38^ For the construction of RNAP and ribosome tagging strain, the upstream and downstream flanking regions of *rpoC2* (*Synpcc7942*_*1524*) and *rpsB* (*Synpcc7942*_*2530*), along with the e*yfp*-Gm^r^ cassette, were amplified and fused. A 5-amino acid linker was incorporated between the C-terminus of each target protein (RpoC2/RpsB) and eYFP to minimize steric interference and ensure proper fusion protein folding, as established in prior studies.^40^^,^^41^ These cassettes were cloned into pEASY-Blunt, generating plasmids pTZ105 and pTZ104, respectively (Table S1). Transformation of pTZ105 and pTZ104 into *S. elongatus* yielded strains RpoC2-eYFP and RpsB-eYFP (Figure S1 and Table S1). For the construction of *kaiC-*null strain, the *kaiC* locus (*Synpcc7942*_*1216*) was replaced with a kanamycin resistance cassette (*Km*^*r*^) from pRL446 using pJET1.2-mediated assembly (ClonExpress MultiS Kit, Vazyme) to generate pWL022, followed by transformation with pTZ105 or pTZ104 to produce strain RpoC2-eYFP (Δ*kaiC*) and strain RpsB-eYFP (Δ*kaiC*) (Table S1). All plasmids were sequence-verified before transformation using established protocols.^42^

#### Circadian rhythm entrainment and inhibitor experiments

To entrain circadian rhythms, cultures were subjected to a 12-h dark period, which has been shown to reset the *S. elongatus* circadian clock.^12^^,^^43^ For inhibitor experiments, Rif (Solarbio) and Cm (Solarbio) were added uniformly 4 h after the onset of illumination to resume DNA replication and ensure normal transcription and translation processes. Both antibiotics were used at a final concentration of 200 μg/mL. Rif and Cm are well-established tools with well-characterized mechanisms of action in prokaryotes. In *S. elongatus*, previous studies have demonstrated that 200 μg/mL Rif inhibits >95% of RNA synthesis,^27^^,^^28^^,^^29^ and 200 μg/mL Cm effectively blocks protein synthesis.^31^^,^^32^ As we used the same cyanobacterial strain and identical inhibitor concentrations as those reported in these previous studies, it is reasonable to conclude that inhibition was effective under our imaging conditions. To assess cell viability following drug treatment, we conducted complementary growth rate analysis and gradient-dilution spot assays. Drug-treated cells were resuspended in fresh BG11 medium (initial OD_730_ = 0.1), cultured under continuous illumination at 30°C. OD_730_ was recorded at 24-h intervals for 120 h. For gradient-dilution viability assessment, cells were serially diluted (10^−1^ to 10^−6^) in BG11 medium, with 10 μL aliquots spotted onto BG11 agar plates. Plates were incubated at 30 °C under constant light for 7 days.

#### Western blot analysis

Total protein extraction from cyanobacterial cells was performed as previously described.^38^ The expression of free eYFP and eYFP-tagged proteins was verified by western blot analysis. Fifty micrograms of total protein were separated by SDS-PAGE, and immunoblotting was conducted following the protocol described by Zhu et al.^44^ A mouse monoclonal anti-GFP-tag antibody (TransGen, China) was used as the primary antibody, followed by incubation with a goat anti-mouse lgG/AP antibody (Thermo Fisher, USA) as the secondary antibody. The protein bands were visualized using BCIP/NBT solution (AMRESCO, USA).

#### Laser scanning confocal microscopy

Confocal microscopy was used because its optical sectioning capability minimizes interference from strong photosynthetic autofluorescence and out-of-focus signals, enabling accurate localization of fluorescently labeled ribosomes and nucleoids in small cyanobacterial cells. At each time point, 1 mL of the *S*. *elongatus* culture was harvested by centrifugation (10,000 × g, 2 min, 25 °C) and re-suspended in 100 μL of sterile deionized water. DNA staining was performed at 30 °C for 15–30 min using 4′,6-diamidino-2-phenylindole (DAPI; Solarbio) at a final concentration of 100 ng/mL. Stained cells were mounted on a microscope slide and a coverslip (Citoglas, China) for imaging. Live-cell images were acquired using an inverted laser scanning confocal microscope (FV1000, Olympus, Japan) equipped with a 100× oil-immersion objective (NA 1.40) and FV10-ASW 4.0 software. DAPI-stained DNA was excited at 405 nm, and emission was collected at 425–465 nm (Blue channel). eYFP-tagged RNAPs and ribosomes were excited at 488 nm, with emission collected at 500–559 nm (Green channel). Cellular autofluorescence was excited at 559 nm, and emission was collected at 575–675 nm (Red channel). Images were captured at a resolution of 1024 × 1024 pixels. The scan speed was set to 20.0 μs/pixel, and the zoom factor was set to 1.6 ×.

#### Short-axis fluorescence intensity profiling

To analyze the spatial redistribution of ribosomes relative to thylakoid membranes (TMs), short-axis fluorescence intensity profiles were generated following established methodologies.^45^ Line scans, conducted perpendicular to the cellular long axis, were performed using ImageJ to extract fluorescence intensities from eYFP-tagged ribosomes and TM autofluorescence, primarily from phycobiliproteins. The signals were then normalized to their peak intensity values and averaged across more than 50 cells per condition. Ribosome-TM spatial relationships were assessed by measuring peak-to-peak distances to evaluate colocalization or spatial segregation.

#### Quantification of overlap coefficient and CI

The overlap coefficient was used to quantify the spatial colocalization between chromosomal DNA and RNAPs or ribosomes,^46^ while the compaction index (CI) was used to evaluate the degree of chromosomal DNA and RNAP condensation.^12^

Quantification of the overlap coefficient was performed using approximately 100 cells from three biological replicates. The overlap coefficient was calculated with ImageJ software (version 1.8.0, Wayne Rasband, USA) using the following formula:

$$r=\frac{\sum_{i}R_{i}\times G_{i}}{\sqrt{\sum_{i}{\left(R_{i}\right)}^{2}\times \sum_{i}{\left(G_{i}\right)}^{2}}}$$

where *R* and *G* represent the pixel intensity values in channel 1 (DNA) and channel 2 (RNAP or ribosome), respectively.^46^ The *R* value ranges from 0 to 1. *r* = 0 indicates complete exclusion, while *r* = 1 indicates complete colocalization. Intermediate values reflect partial overlap. This method is relatively insensitive to signal intensity fluctuations during image acquisition.^46^ To quantify the compaction index (CI) of chromosomal DNA and RNAP, the autofluorescence signal was first processed by enhancing its intensity to three times the average background level over a small number of pixels to define cell boundaries.^12^ The DNA or RNAP signal was then analyzed for spatial overlap with this processed autofluorescence channel using the same overlap coefficient calculation. The resulting R value was defined as the CI of either DNA or RNAP. CI values range from 0 to 1. CI = 1 indicates a uniform signal distribution across the entire cell, while CI = 0 reflects a highly compacted signal with no pixels captured.

#### Cosine regression analysis of rhythmicity

To quantitatively assess circadian rhythmicity, time-series data were fitted to a 24-h cosine model using least-squares regression. For each dataset, the following function was used:

y(t) = a·cos(2πt/24) + b·sin(2πt/24) + c, where t represents time (in hours), and a, b, and c are fitted coefficients. This formulation allows flexible fitting of oscillatory signals with arbitrary phase and amplitude, and is mathematically equivalent to a cosine function with a defined amplitude and phase (i.e., y = baseline + amplitude × cos (2πt/24 + φ)).

Regression analysis was performed independently for each biological replicate. The goodness-of-fit of the model was evaluated using the coefficient of determination (R^2^), which reflects the proportion of variance explained by the 24-h periodic model. Statistical significance (*p*-value) was obtained from the regression analysis associated with the fitted model, reflecting whether inclusion of oscillatory terms significantly improves the fit compared to a non-oscillatory baseline.

A threshold of *p* < 0.05 was used to define statistically significant rhythmicity. In addition, R^2^ values were used to assess the strength of the oscillatory fit, with higher values indicating stronger conformity to a 24-h periodic pattern, whereas low R^2^ values (e.g., <0.4) indicate weak or absent periodic structure.

### Quantification and statistical analysis

Quantitative analysis was performed using ImageJ software, as described in the “Quantification of the overlap coefficient and compaction index (CI)” section of the Methods Details. Statistical analysis was conducted using GraphPad Prism software, as described in the “Cosine regression analysis of rhythmicity” section of the method details.

### Additional resources

No additional external resources, datasets, or code repositories were used or developed for this study.
