# Supplementary material for: KaiC-dependent circadian positioning of RNA polymerase and ribosome in cyanobacteria
Source: iScience. 2026 Jun 27;29(7):116630. doi: 10.1016/j.isci.2026.116630 (PMC13378304; doi:10.1016/j.isci.2026.116630)
Supplement: Document S1. Figures S1–S8 and Tables S1–S3 [file mmc1.pdf]

iScience, Volume *29*

Supplemental information

KaiC-dependent circadian positioning of RNA polymerase and ribosome in cyanobacteria

Lina Wang, Cuncun Qiao, Chi Zhao, Tao Zhu, and Xuefeng Lu

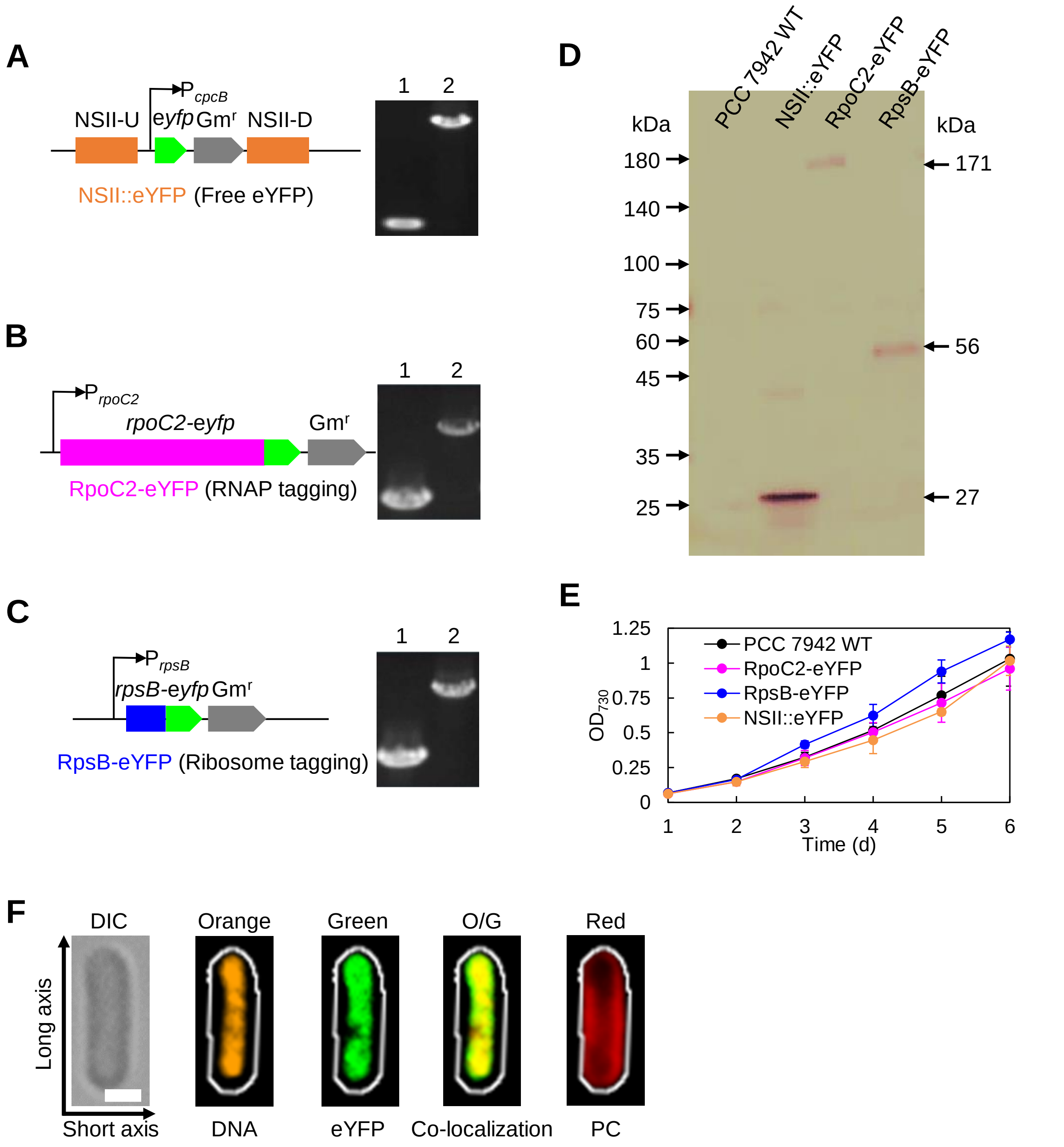

Figure S1. Construction and characterization of strains NSII::eYFP, RpoC2-eYFP, and RpsB-eYFP. (A) Schematic diagram of the *eyfp* gene driven by P_{cpcB} integrated into the neutral site II (NSII) of the *S. elongatus* genome (left). Agarose gel electrophoresis (AGE) of PCR products amplified using primers flanking the NSII locus (right). Lane 1, *S. elongatus* WT (0.3 kb); Lane 2, NSII::eYFP strain (2.8 kb). (B) Schematic diagram of the in-frame fusion of the *eyfp* gene to the 3'-end of the *rpoC2* (left). The AGE of PCR products amplified using primers targeting the internal region of *rpoC2* and its 3'-flank sequence (right). Lane 1, *S. elongatus* WT (1.9 kb); Lane 2,

RpoC2-eYFP strain (3.8 kb). (C) Schematic diagram of the in-frame fusion of *eyfp* to the 3′ end of *rpsB* (left). AGE of PCR products amplified using primers targeting the internal region of *rpsB* and its 3′ flank (right). Lane 1, *S*. *elongatus* WT (2.0 kb); Lane 2, RpsB-eYFP strain (3.9 kb). (D) Western blot analysis confirming the correct integration and expression of eYFP in the NSII::eYFP, RpoC2-eYFP, and RpsB-eYFP strains. *S. elongatus* WT served as a negative control. Total protein extracts were probed with an anti-GFP monoclonal antibody. The data shown are representative of technical triplicates. (E) Growth curves of strains *S. elongatus* WT, NSII::eYFP, RpoC2-eYFP, and RpsB-eYFP. Results represent three biological replicates. (F) Representative laser scanning confocal images of *S. elongatus* cells. DIC: Differential interference contrast. Orange pseudo-color: chromosomal DNA (DAPI-stained; acquired in blue emission channel); Green: free eYFP or eYFP-tagged RNAP/ribosomes (acquired in green emission channel). Overlay (O/G): spatial co-localization of DNA and RNAP/ribosomes; Red: autofluorescence of phycobiliproteins (acquired in red emission channel). Cell long and short axes are indicated in the DIC image. Scale bar = 1 μm.

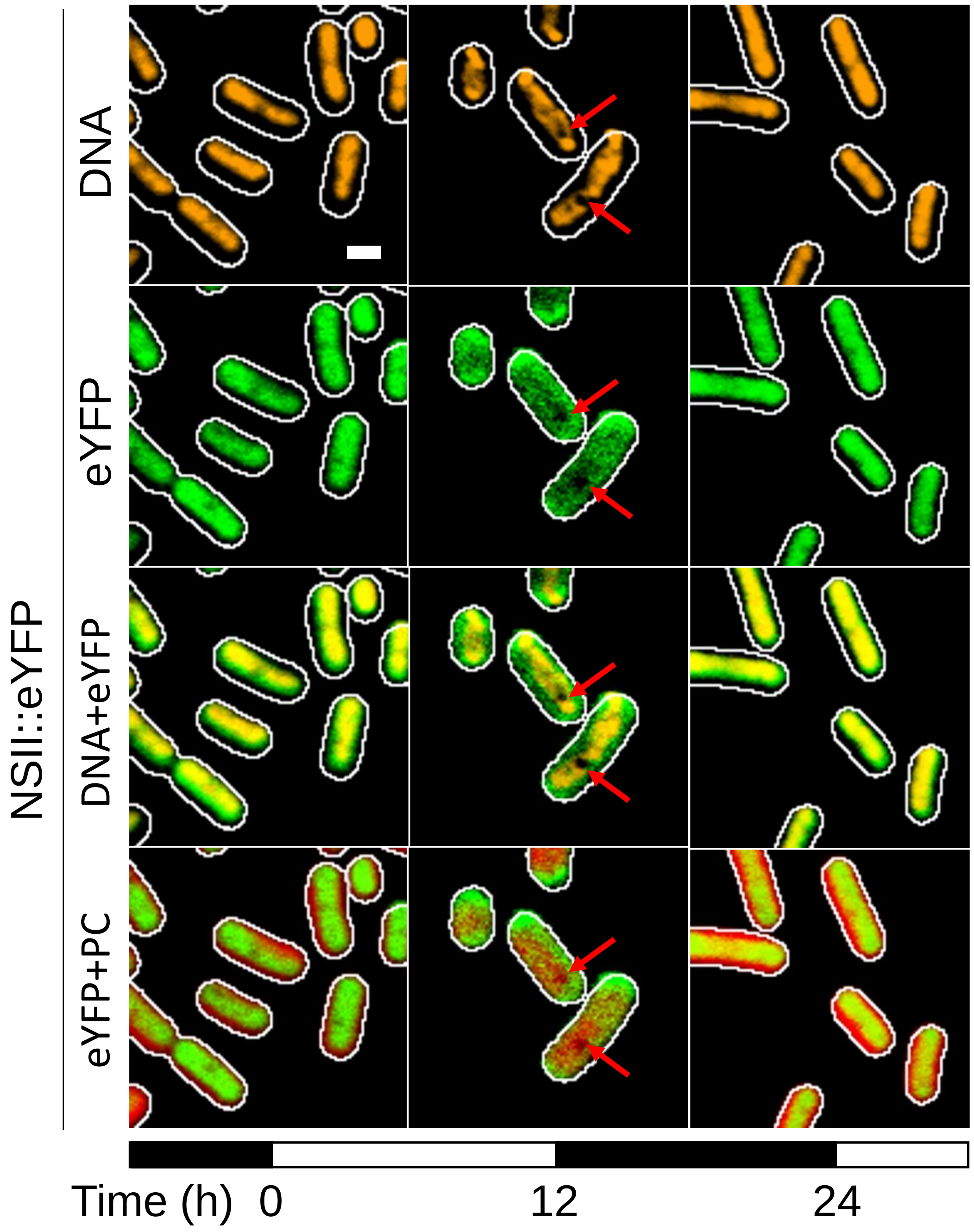

Figure S2. Confocal microscopy images showing the intracellular distribution of free eYFP in *S. elongatus* under the LD cycle. *S. elongatus* cultures growing under constant light were first subjected to 12 h of darkness to reset their circadian rhythms. The cultures were then transferred to light, and this time point was defined as time 0. The timeline under the images indicates dark (black) and light (white) phases. Images at each time point represent >90% of the observed cell population. Occasional dark regions (indicated by red arrows) during DNA condensation may correspond to carboxysome locations. Orange: DAPI-stained DNA; Green: free eYFP; Red, autofluorescence from phycobiliproteins. Scale bars = 1 μm.

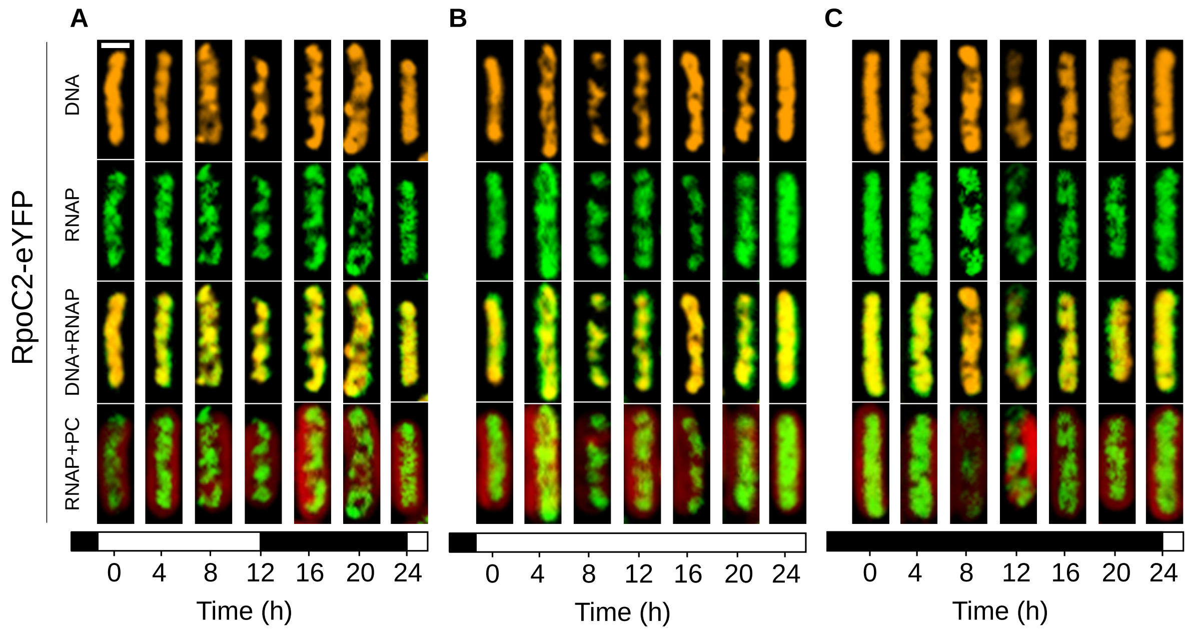

Figure S3. Imaging of individual *S. elongatus* cells showing chromosomal DNA and RNAPs transitioning between diffuse and condensed states under LD, LL, and DD conditions (4-h intervals). (A-C) Confocal microscopy images of representative *S. elongatus* RpoC2-eYFP cells sampled at indicated time points during the first cycle of LD (A), LL (B), and DD (C) conditions. (A): Zeitgeber Time (ZT), with ZT 0 = lights-on (dawn), ZT 12 = lights-off (dusk). (B) and (C): Circadian Time (CT), with CT 0 = subjective dawn and CT 12 = subjective dusk. Cultures were entrained by 12 h darkness before release at ZT 0 (LD) or CT 0 (LL/DD). Timepoints (4-h intervals) correspond to indicated ZT/CT values. Each image represents the typical pattern observed in over 90% of cells at that time point. Orange: DAPI-stained DNA; Green: RNAPs; Red, autofluorescence from phycobiliproteins. Scale bars = 1 μm.

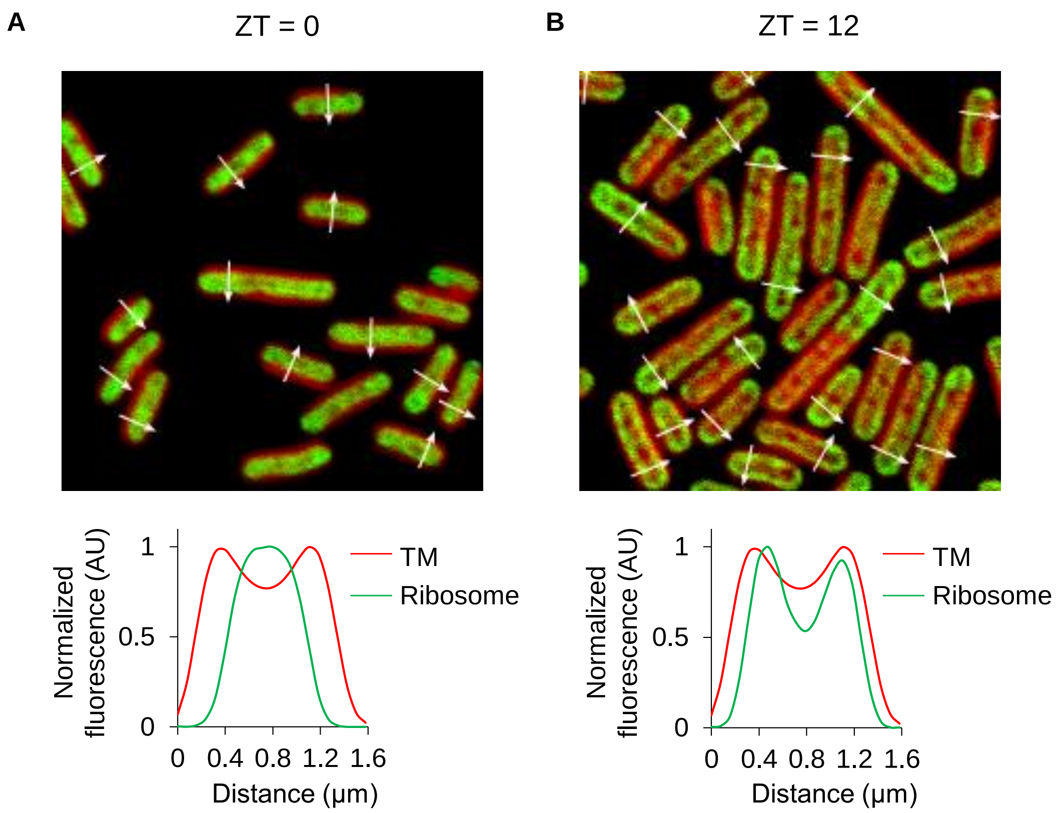

Figure S4. Short-axis fluorescence intensity profiles of ribosomes and thylakoid membranes under LD condition. (A) Merged confocal imaging of ribosome and thylakoid membranes at ZT 0 (top), and corresponding short-axis fluorescence intensity profiles averaged from more than 50 cells (bottom). (B) Merged confocal images at ZT 12 (top), and corresponding fluorescence intensity profiles from more than 50 cells (bottom). TM: thylakoid membranes; AU: arbitrary units. Green: ribosomes; Red, autofluorescence from phycobiliproteins.

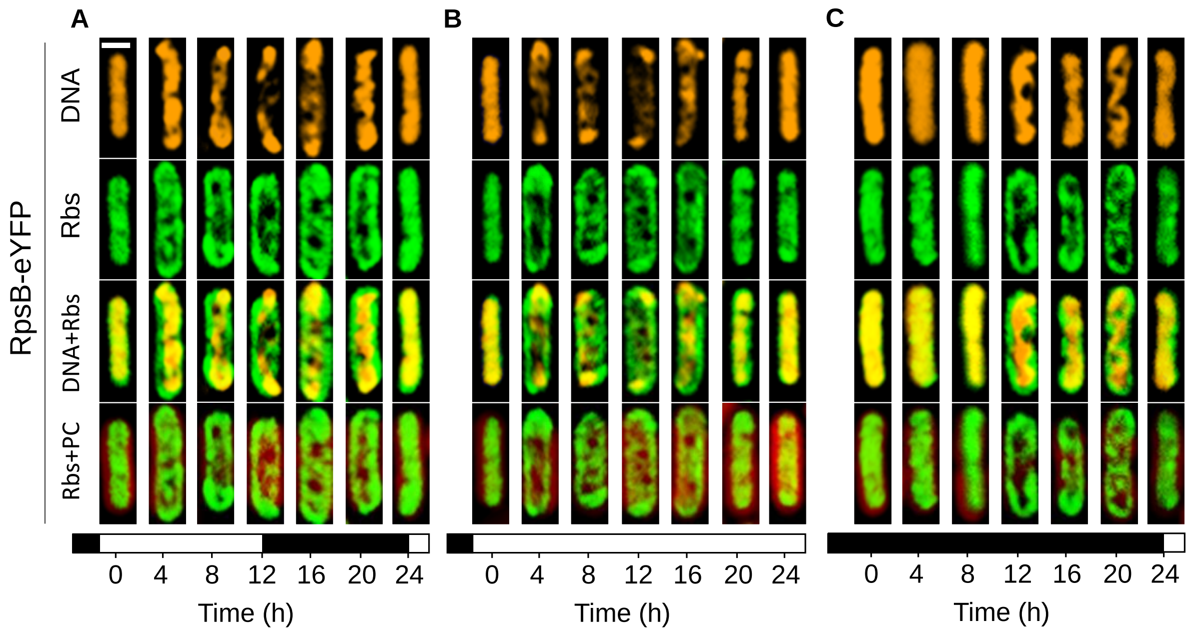

Figure S5. Imaging of individual *S. elongatus* cells showing the dynamic condensation of chromosomal DNA and exclusion of ribosomes toward thylakoid membrane regions under LD, LL, and DD conditions (4-h intervals). (A-C) Confocal microscopy images of representative *S. elongatus* RpsB-eYFP cells sampled at indicated time points during the first cycle of LD (A), LL (B), and DD (C) conditions. (A): Zeitgeber Time (ZT), with ZT 0 = lights-on (dawn), ZT 12 = lights-off (dusk). (B) and (C): Circadian Time (CT), with CT 0 = subjective dawn and CT 12 = subjective dusk. Cultures were entrained by 12 h darkness before release at ZT 0 (LD) or CT 0 (LL/DD). Timepoints (4-h intervals) correspond to indicated ZT/CT values. Each image represents the typical pattern observed in over 90% of cells at that time point. Orange: DAPI-stained DNA; Green: RNAPs; Red, autofluorescence from phycobiliproteins. Scale bars = 1 μm.

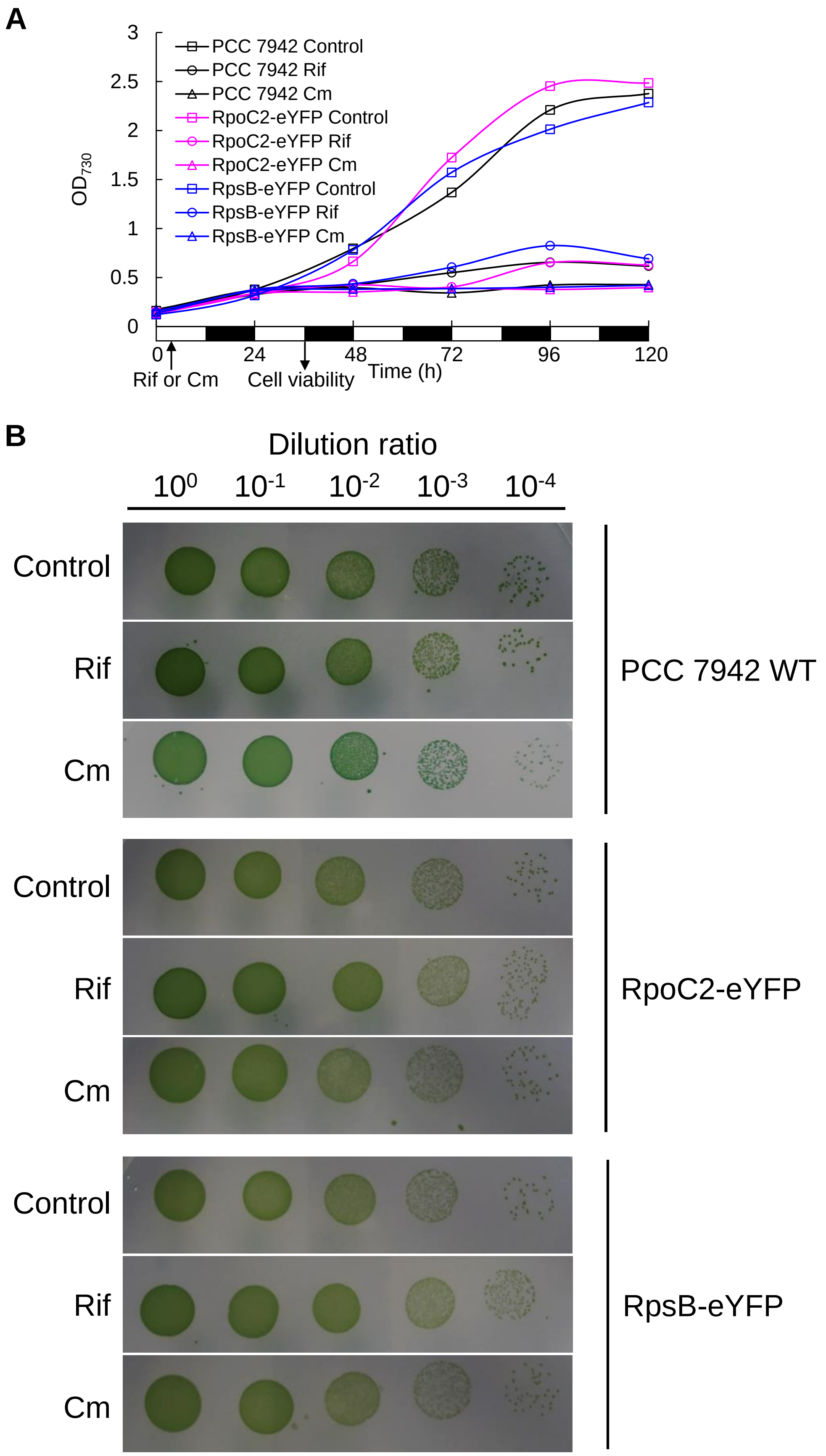

Figure S6. Growth curves and cell viability test under the treatment of Rif (200 µg/ml) and Cm (200 µg/ml). (A) Growth curves of strains *S. elongatus* WT,

RpoC2-eYFP, and RpsB-eYFP strains treated with either no drugs, rifampicin (Rif, 200 μg/mL), or chloramphenicol (Cm, 200 μg/mL). To entrain the circadian rhythm of these strains, cultures were first subjected to a 12-hour dark period. The cultures were then transferred to light, and this time point was defined as time 0. Subsequently, samples were collected at 12-hour intervals for 72 h. Drug treatments were administered at ZT 4. The timeline (black/white bars) indicates dark and light phases, respectively. (B) Cell viability assay at time 36 h. Cells were adjusted to an OD_{730} of 2 × 10^{-5} and serially diluted (10^{-1} to 10^{-4}) before spotting onto BG-11 agar plates. Plates were incubated at 30 °C under constant illumination for 7 days.

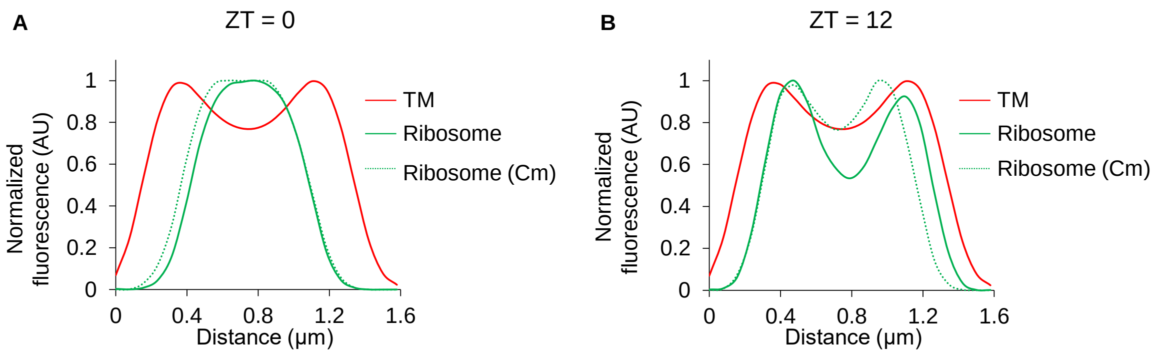

Figure S7. Short-axis fluorescence intensity profiles of ribosomes, thylakoid membranes, and Cm-treated ribosomes. (A) Fluorescence intensity profiles of ribosomes, thylakoid membranes, and Cm-treated ribosomes at ZT 0, averaged from more than 50 cells. (B) Fluorescence intensity profiles of the same components at ZT 12, averaged from more than 50 cells. TM: thylakoid membranes; AU: arbitrary units.

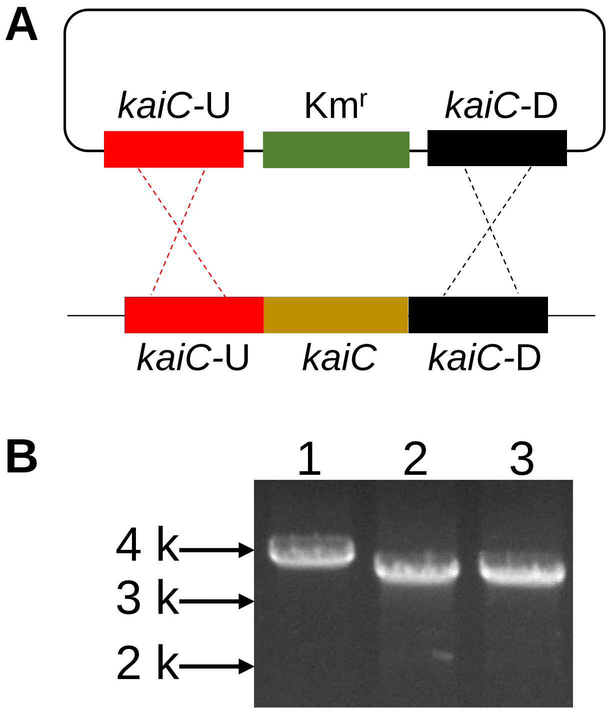

Figure S8. Construction and verification of RpoC2-eYFP (Δ*kaiC*) and RpsB-eYFP (Δ*kaiC*) strains. (A) Schematic diagram illustrating the deletion of the *kaiC* gene in *S. elongatus* via double homologous recombination. (B) AGE image of PCR products amplified using primers flanking the *kaiC* locus. Lane 1: *S. elongatus* WT (3.7 kb); Lane 2: strain RpoC2-eYFP (Δ*kaiC*) (3.3 kb); Lane 3: strain RpsB-eYFP (Δ*kaiC*) (3.3 kb).

Table S1. Strains, plasmids, and primers used in this work. Related to STAR METHODS.

	Derivation and/or relevant characteristics[a]	Sources
**Plasmids**		
pLAU53	Ap^r, plasmid containing the e*yfp* cassette	CGSC
pEASY-Blunt Simple	Ap^r, cloning vector	TransGen
pJET1.2 Blunt vector	Ap^r, cloning vector	Thermo Fisher
pMH005	Gm^r, plasmid containing the Gm^r cassette	Laboratory strain
pRL446	Ap^r Km^r, plasmid containing the Km^r cassette	Prof. Xudong Xu
pQL224n	Ap^r, *S. elongates* neutral site 2 (NSII) integration vector	Laboratory strain
pQL233	Ap^r Gm^r, pQL224n derivative containing P_{cpcB}-*eyfp*-Gm^r expression cassette	This work
pTZ105	Ap^r Km^r Gm^r, pEASY-Blunt Simple vector derivative for chromosomal insertion of "*rpoC2*-e*yfp*-Gm^r" fusion at native locus	This work
pTZ104	Ap^r Km^r Gm^r, pEASY-Blunt Simple vector derivative for chromosomal insertion of "*rpsB*-*eyfp*-Gm^r" fusion at native locus	This work
pTZ110	Ap^r Km^r Sp^r, pEASY-Blunt Simple vector derivative used to delete *ftsZ* with Sp^r cassette	This work
pWL022	Ap^r Km^r, pJET 1.2 Blunt vector derivative used to delete *kaiC* with Km^r cassette	This work
**Strains**		
S. elongates	*Synechococcus elongatus* PCC 7942 wild type	Laboratory strain
NSII::eYFP	NSII::P_{cpcB}-e*yfp*-Gm^r	This work
RpoC2-eYFP	*S. elongates* with chromosomal "*rpoC2*-e*yfp*" fusion	This work
RpsB-eYFP	*S. elongates* with chromosomal "*rpsB*-e*yfp*" fusion	This work
RpoC2-eYFP (Δ*kaiC*)	RpoC2-eYFP strain with *kaiC* deletion	This work
RpsB-eYFP (Δ*kaiC*)	RpsB-eYFP strain with *kaiC* deletion	This work
**Primers (5′→3′)**		
**For NSII::eYFP**		
P_{cpcB}-1	CTACAGCCTGGGTTCCTCATG	
P_{cpcB}-2	GTCGAGAACAGGAGACTGGTTGAGTGAGCAAGGGCGAGGAGC	
e*yfp*-1	GTCGAGAACAGGAGACTGGTTGAGTGAGCAAGGGCGAGGAG	
e*yfp*-2	GTCCCCTTATACACAAGGACTAAGAGTGATCCCGGCGGCG	
Gm-1	CGCCGCCGGGATCACTCTTAGTCCTTGTGTATAAGGGGAC	
Gm-2	GGCCGGGAAGCCGATCTCGG	
**For RpoC2-eYFP**		
rpoC2-U1	CCTCGAGGCTCGTAAACCCA	
rpoC2-U2	CTCACCAACTCGAGGATGTCGTCTTCGTCAACATCCACCA	
rpoC2yfpGm-1	TGGTGGATGTTGACGAAGACGACATCCTCGAGTTGGTGAG	
rpoC2yfpGm-2	ACCGCACCGGCAGAAAGCACGGCCGGGAAGCCGATCTCGG	
rpoC2-D1	CCGAGATCGGCTTCCCGGCCGCAAGCGATCGCGGTGGAGT	
rpoC2-D2	GTCAGCAGTTCCAGCGCTAT	
**For RpsB-eYFP**		
rpsB-U1	GGCGCGTTGCTTCAGGCCAA	
rpsB-U2	CTCACCAACTCGAGGATGTCTGCTTCGTCGGCGTCGTCTT	
RpsByfpGm-1	AAGACGACGCCGACGAAGCAGACATCCTCGAG TTGGTGAG	
RpsByfpGm-2	ACTCCACCGCGATCGCTTGCGGCCGGGAAGCCGATCTCGG	
rpsB- D1	CCGAGATCGGCTTCCCGGCCGCAAGCGATCGCGGTGGAGT	

rpsB- D2	GTCAGCAGTTCCAGCGCTAT	
**For △*kaiC***		
KaiC-U1	GTTTTTCAGCAAGATTTCAGGGCAGTTGTTCAGCAG	
KaiC-U2	CACCGCCTACATACCTCGCTCTTAAAAGAGGGTGAAGTCA	
KaiC-Km-1	AGCGAGGTATGTAGGCGGTGCT	
KaiC-Km-2	TACCTAAGCGCGATCGCTGGGGAAATGTGCGCGGAACCCCT	
KaiC-D1	CCAGCGATCGCGCTTAGGTATT	
KaiC-D2	ATCTTCTAGAAAGATCCGCTGATATGGCTCCTCGAT	

[a]Ap^r, Km^r and Gm^r indicate ampicillin, kanamycin and gentamicin resistance genes, respectively.

Table S2. Manders' colocalization coefficients for RNAP (RpoC2-eYFP) and ribosomes (RpsB-eYFP) under diffuse and compacted nucleoid conditions, related to Figures 1A and 3A.

Strain	ZT	M1 (DNA→target)	M2 (target→DNA)
RpoC2-eYFP (RNAP)	ZT 0 (diffuse)	0.999	0.983
RpoC2-eYFP (RNAP)	ZT 12 (compacted)	0.999	0.998
RpsB-eYFP (Ribosome)	ZT 0 (diffuse)	0.999	0.992
RpsB-eYFP (Ribosome)	ZT 12 (compacted)	0.857	0.843

M1 represents the fraction of DNA signal overlapping with the target (RNAP or ribosome); M2 represents the fraction of target signal overlapping with DNA. Values range from 0 to 1, where values close to 1 indicate complete colocalization, and values close to 0 indicate no colocalization.

Table S3. Cosine regression analysis of rhythmicity under LD conditions

Parameter	Strains	Genotypes	R^2	P value	Interpretation
CI (DNA)	RpoC2-eYFP	WT	0.7483	0.0025	Signification rhythm
	RpoC2-eYFP (Δ*kaiC*)	Δ*kaiC*	0.026	0.46	No rhythm
CI (RNAP)	RpoC2-eYFP	WT	0.4694	0.025	Strong rhythm
	RpoC2-eYFP (Δ*kaiC*)	Δ*kaiC*	0.0754	0.207	No rhythm
Overlap (DNA/RNAP)	RpoC2-eYFP	WT	0.0069	0.8	No rhythm
	RpoC2-eYFP (Δ*kaiC*)	Δ*kaiC*	0.3899	0.056	No rhythm
CI (DNA)	RpsB-eYFP	WT	0.8224	0.005	Signification rhythm
	RpsB-eYFP (Δ*kaiC*)	Δ*kaiC*	0.154	0.062	No rhythm
Overlap (DNA/Ribosome)	RpsB-eYFP	WT	0.9234	0.001	Signification rhythm
	RpsB-eYFP (Δ*kaiC*)	Δ*kaiC*	0.2924	0.135	No rhythm

Rhythmicity was assessed using the p-value and the coefficient of determination (R^2). The goodness-of-fit of the oscillatory model was evaluated using R^2 (range: 0–1), where higher values indicate stronger conformity to a 24-h periodic pattern, and lower values (e.g., $R^2 < 0.4$) suggest weak or absent periodic structure. A p-value < 0.01 indicates statistically significant rhythmicity, values between 0.01 and 0.05 indicate strong rhythmicity, and $p > 0.05$ indicates no detectable rhythmicity.
